# Sustainable Hybrid Flame-Retardant Architectures for LDPE: Effects of Waste-Derived Cellulose, SiO_2_, and APP/ADA

**DOI:** 10.3390/polym18202566

**Published:** 2026-10-18

**Authors:** Antelmo R. Y. Ruiz-Martínez, Edgar Nazareo Cabrera-Álvarez, Luciano da Silva, Arxel de León, Ramón Diaz de León, David Contreras-López

**Affiliations:** 1 Centro de Investigación en Química Aplicada (CIQA), Blvd. Enrique Reyna #140, Col. San José de Los Cerritos, Saltillo 25294, COAH, Mexico; antelmo.ruiz@ciqa.edu.mx (A.R.Y.R.-M.); luciano.dasilva@ciqa.edu.mx (L.d.S.); ramon.diazdeleon@ciqa.edu.mx (R.D.d.L.); 2 Secretaría de Ciencia, Humanidades, Tecnología e Innovación (SECIHTI)-Centro de Investigación en Química Aplicada (CIQA), Unidad Monterrey, Alianza sur #204, Parque de Investigación e Innovación Tecnológica (PIIT), km 10 Autopista Internacional Mariano Escobedo, Apodaca 66628, NL, Mexico; 3 Secretaría de Ciencia, Humanidades, Tecnología e Innovación (SECIHTI)-Centro de Investigación en Química Aplicada (CIQA), Blvd. Enrique Reyna #140, Col. San José de Los Cerritos, Saltillo 25294, COAH, Mexico; arxel.deleon@ciqa.edu.mx; 4 Departamento de Ingeniería Química, Universidad de Guanajuato, Noria Alta s/n, Guanajuato 36050, GTO, Mexico; david.contreras@ugto.mx

**Keywords:** LDPE, flame retardant grenade, waste-derived cellulose, ammonium polyphosphate, SiO_2_, azodicarbonamide

## Abstract

This study evaluates the flame retardant efficiency of hybrid systems based on natural cellulose extracted from agricultural wastes (pecan nutshell and guayule), combined with azodicarbonamide (ADA), porous silicon dioxide (SiO_2_) scaffolds, and ammonium polyphosphate (APP), incorporated into low-density polyethylene (LDPE). The waste-derived cellulose fractions were isolated via alkaline extraction and steam explosion, yielding microfibrils that preserved native lignin fractions. Porous SiO_2_ particles were modified through the adsorption of ADA and APP, which in turn decorated the surface of the cellulose particles. Hybrid LDPE composites containing 20 wt% of total additives were melt-compounded and characterized. Thermogravimetric analysis (TGA) revealed an early intumescent activation that promoted robust charring, achieving final char residue yields up to 9.5 wt% for the pecan nutshell composite. Horizontal UL-94 and cone calorimeter tests demonstrated the complete elimination of hazardous flaming melt-dripping across all formulated hybrid systems, accompanied by a significant reduction in the burning rate to 18.3 mm/min and a 45% reduction in PHRR for the pecan nutshell-derived compound, consistent with a protective contribution of the multifunctional particles in the condensed phase, significantly outperforming its commercial nanocellulose counterparts. Cone calorimetry highlighted a drastic flattening of the peak heat release rate (PHRR) kinetics of composites. FTIR, TEM and TGA analyses confirmed the formation of a APP-ADA-loaded SiO_2_ particle scaffold (“grenade-like” morphology), with combustion-residue FTIR and SEM/EDS supporting a protective contribution in the condensed phase.

## 1. Introduction

Low-density polyethylene (LDPE) is a highly versatile thermoplastic polymer extensively utilized in packaging, cable insulation, and agricultural films due to its excellent flexibility, processability, and chemical resistance. However, its aliphatic hydrocarbon structure renders it inherently highly flammable. During combustion, LDPE exhibits severe flaming melt-dripping, a phenomenon that accelerates fire propagation and severely restricts its application in environments with stringent fire safety regulations [1,2]. Consequently, developing environmentally sustainable, halogen-free flame-retardant (FR) strategies for LDPE remains a critical industrial and academic priority [3]. 

Intumescent flame-retardant (IFR) systems represent a highly effective strategy for polymers, operating primarily via condensed-phase mechanisms. A typical IFR formulation relies on the synergistic interaction of an acid source, a carbon source, and a blowing agent, which upon thermal degradation form a swollen, multi-cellular carbonaceous shield. This barrier impedes heat transfer, oxygen diffusion, and the escape of combustible volatile fuel. Ammonium polyphosphate (APP) is the benchmark acid source; it catalyzes the dehydration of hydroxyl-rich compounds to form carbon double bonds, ultimately leading to charring. Nevertheless, when dispersed alone within the highly nonpolar LDPE matrix, APP exhibits poor interfacial compatibility. This phase separation frequently leads to the formation of structurally weak, discontinuous char layers. Indeed, the limited interfacial compatibility and the necessity for excessively high APP loadings to achieve desired flame retardancy are pervasive challenges not only in nonpolar polyolefins but broadly across various polymer matrices, including polyurethanes [4,5].

To overcome these structural limitations, this work introduces the concept of a Hybrid Flame-Retardant Grenade (FRG) architecture. The FRG approach moves beyond the random dispersion of isolated additives by rationally integrating the active IFR components—APP (acid source), azodicarbonamide (ADA, blowing agent), a renewable cellulose (carbon source) and silica (SiO_2_, scaffold)—in a cooperative functional network. Previous studies suggest that, during thermal decomposition, SiO_2_ and APP may contribute to the formation of protective inorganic structures [6,7,8]. ADA is considered a potential blowing agent whose decomposition may contribute to residue expansion. Furthermore, the incorporation of SiO_2_ nanoparticles enhances the structural integrity and thermal stability of the protective char [9,10]. These proposed contributions are consistent with the development of an intumescent protective residue.

Within this hybrid FRG architecture, cellulosic materials serve as renewable carbon precursors. Highly purified commercial cellulose nanocrystals (CNC) and cellulose nanofibrils (CNF) possess high specific surface areas and strong mechanical reinforcing capabilities that favor dense char formation [11,12]. Nonetheless, their energy-intensive extraction and high production costs limit widespread industrial scalability. Conversely, abundant agro-industrial residues, such as pecan nut shells (PNS) and guayule bagasse (GC), present a sustainable alternative. Unlike highly purified nanocelluloses, these minimally processed lignocellulosic fibers retain fractions of lignin. From a thermodynamic perspective, the highly cross-linked, aromatic structure of lignin undergoes earlier cyclization and aromatization at elevated temperatures compared to purely aliphatic cellulose. This inherent property provides an additional functional advantage, since residual lignin can contribute to char formation, as previously reported [13].

Despite the growing interest in cellulose-based and carbon-based FR materials [14,15], systematic evaluations directly comparing highly refined nanocelluloses (CNC, CNF) against lignin-containing microfibrillated agricultural residues (PNS, GC) within a complex, hybrid intumescent system are scarce. Establishing how the interplay among cellulose source and morphology modifies the thermal degradation kinetics and final fire behavior of LDPE is essential to validate the use of upcycled biomass in advanced composites.

Accordingly, this study systematically evaluates the proposed FRG architecture by comparing four distinct cellulosic scaffolds integrated with an APP/ADA/SiO_2_ system. Through melt compounding into LDPE, the structural integrity, thermal stability, crystallization behavior, and fire performance of the resulting composites were comprehensively characterized using FTIR, XRD, TEM, TGA, DSC, UL-94, and cone calorimetry. This comparative framework aims to elucidate the structure-property relationships governing biomass-driven intumescence, ultimately determining whether raw lignocellulosic waste can effectively replace traditional nanocelluloses as a sustainable carbon sink for high-performance flame-retardant LDPE composites.

## 2. Materials and Methods

### 2.1. Materials

Low-density polyethylene (LDPE EF412 grade, 2 g/10 min, 0.923 g/cm^3^) was supplied by Westlake Chemical (Houston, TX, USA). Phase-II ammonium polyphosphate (APP; Preniphor™ EPFR-APP222H, degree of polymerization *n* ≥ 1000) was provided by Presafer (Qingyuan) Phosphor Chemical Co., Ltd. (Qingyuan, China). Azodicarbonamide (ADA, 99.8%), tetraethyl orthosilicate (TEOS, 99.6%), ammonium hydroxide (NH_4_OH, 28–30 wt% NH_3_), cetyltrimethylammonium bromide (CTAB, 98%), absolute ethanol (99.6%), and all analytical-grade reagents were purchased from Sigma-Aldrich (St. Louis, MO, USA) and used without further purification. Pecan nutshell (PNS) with irregular size was obtained from a local producer from Nuevo León, México. The guayule plant (*Parthenium argentatum*, GLY) was harvested from crops cultivated in the experimental fields at the Center for Research in Applied Chemistry (CIQA). Cellulose nanofibrils (CNF): commercial-grade nanofibrils supplied by Borregaard AS (Sarpsborg, Norway) and used as received. Cellulose nanocrystals (CNC): commercial-grade nanocrystals supplied by CelluForce Inc. (Montreal, QC, Canada) and used as received.

### 2.2. Methods

#### 2.2.1. Synthesis of Porous Amorphous SiO_2_

Amorphous silica (SiO_2_) particles were synthesized using a surfactant-assisted modified Stöber sol–gel method with cetyltrimethylammonium bromide (CTAB) as the structure-directing agent [16]. Distilled water and absolute ethanol were mixed under magnetic stirring, followed by the addition of CTAB and tetraethyl orthosilicate (TEOS). The suspension was homogenized by sonication and maintained under continuous stirring at 50 °C. Subsequently, ammonium hydroxide (NH_4_OH) was added as a basic catalyst to promote the hydrolysis and condensation of TEOS.

After 5 h of reaction, the precipitated silica particles were recovered by centrifugation, washed repeatedly with deionized water and ethanol, and dried at 80 °C for 4 h. The dried material was calcined in air at 600 °C for 6 h to remove the CTAB template [17]. The resulting silica powder (SiO_2_) was gently ground and stored under dry conditions until further characterization and composite preparation.

#### 2.2.2. Extraction of Waste-Derived Cellulosic Scaffolds (PNS and GC)

**Figure 1:**
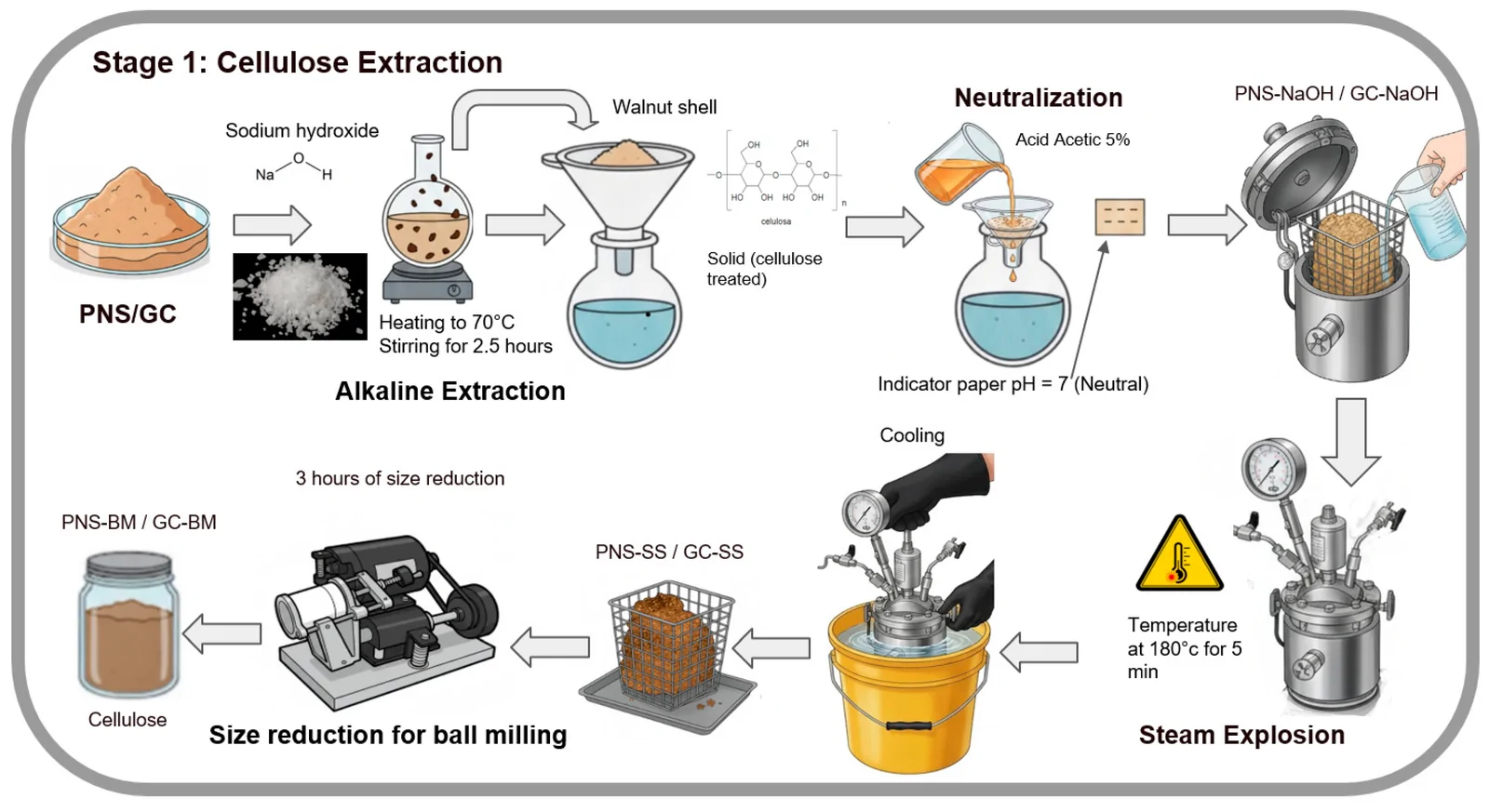
Schematic illustration of the extraction process for waste-derived cellulose. The lignocellulosic biomass was subjected to alkaline treatment, followed by steam explosion and high-energy ball milling to obtain cellulosic powders for subsequent FRG fabrication.

Cellulosic scaffolds were extracted from pecan nutshell (PNS) and guayule bagasse (GC) using a modified alkaline extraction procedure combined with steam explosion as shown in Figure 1. The process was designed to partially remove hemicellulose while preserving a fraction of the native lignin. Raw biomass was ground and treated with a 2 wt% NaOH solution at 80 °C for 2 h under continuous stirring. The treated fibers were washed repeatedly with distilled water and 5 wt% acetic acid until neutral pH was reached and subsequently dried at 60 °C. At this stage, the samples were designated as GC-NaOH and PNS-NaOH. The pretreated material was then subjected to steam explosion at 180 °C (150 psi) for 10 min to promote fiber defibrillation and disruption of the lignocellulosic structure (PNS-SS and GC-SS). After steam explosion, the material was mechanically refined by high-energy ball milling at 300 rpm for a total milling time of 60 min, divided into four cycles of 15 min separated by 2 min cooling intervals to minimize excessive heat accumulation during processing. The resulting cellulosic powders were identified as PNS-BM for the pecan nutshell and GC-BM for the guayule bagasse, and were stored in a desiccator until their use in the fabrication of the hybrid flame-retardant grenade (FRG).

#### 2.2.3. Fabrication of the Hybrid Flame-Retardant Granade (FRG)

**Figure 2:**
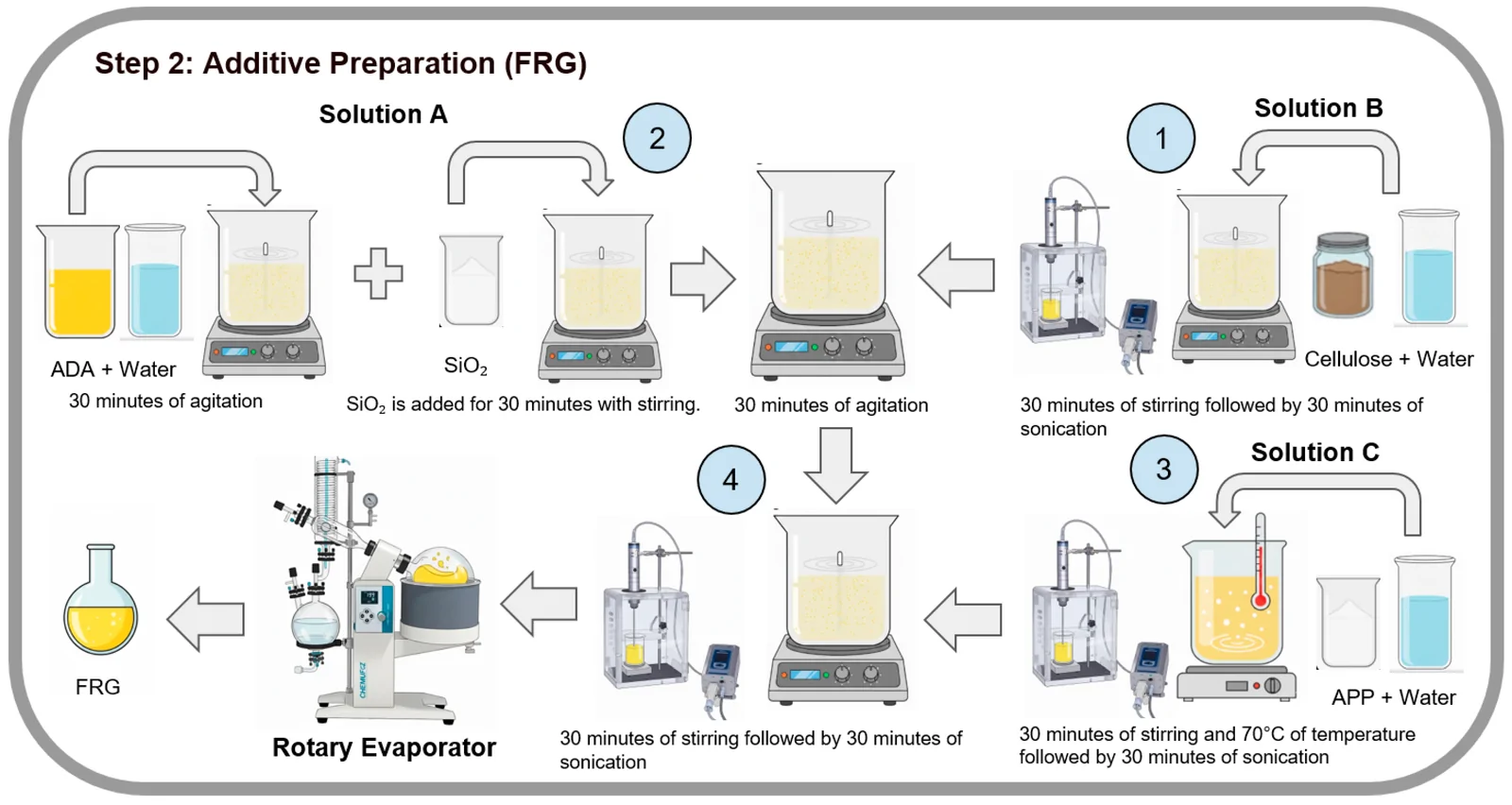
Schematic illustration of the fabrication procedure of the Hybrid Flame-Retardant Grenade (FRG). The process involves preparation of individual cellulose, ADA/SiO_2_, and APP suspensions, followed by sequential mixing, ultrasonication, solvent removal by rotary evaporation, and recovery of the FRG powder.

The Hybrid Flame-Retardant Granade (FRG) was prepared following the same procedure for all four cellulosic scaffolds (PNS-BM, GC-BM, CNC, and CNF) as shown in Figure 2. The formulation was designed using a mass ratio of 5:5:5:1 for APP, cellulose, ADA, and SiO_2_, respectively.

1. Preparation of the cellulose suspension. A total of 7 g of the selected cellulosic scaffold was dispersed in 140 mL of distilled water under mechanical stirring at 850 rpm for 30 min. The suspension was subsequently ultrasonicated using a probe sonicator (Qsonica, 60% amplitude) for 30 min while maintaining the temperature below 25 °C using an ice bath.
2. Preparation of the ADA/SiO_2_ suspension. Separately, 7 g of ADA were dispersed in 720 mL of distilled water at 50 °C under continuous stirring for 30 min. Subsequently, 1.4 g of the synthesized amorphous SiO_2_ particles were added and the suspension was stirred for an additional 30 min. The cellulose suspension was then incorporated into the ADA/SiO_2_ dispersion and homogenized for 30 min.
3. Preparation of the APP suspension. In a separate vessel, 7 g of phase-II ammonium polyphosphate (APP) was dispersed in 140 mL of distilled water under mechanical stirring at 750 rpm for 5 min, followed by ultrasonication (60% amplitude) for 30 min at 25 °C. The APP suspension was then added to the cellulose/ADA/SiO_2_ mixture.
4. FRG recovery. The resulting suspension was homogenized by low-intensity ultrasonication (20% amplitude) at 60 °C for 30 min. Water was removed using a rotary evaporator operated at 60 °C and 170 rpm. The recovered material was vacuum-dried, gently ground into powder, and stored in a desiccator until composite preparation.

#### 2.2.4. Preparation of LDPE/FRG Composites

**Figure 3:**
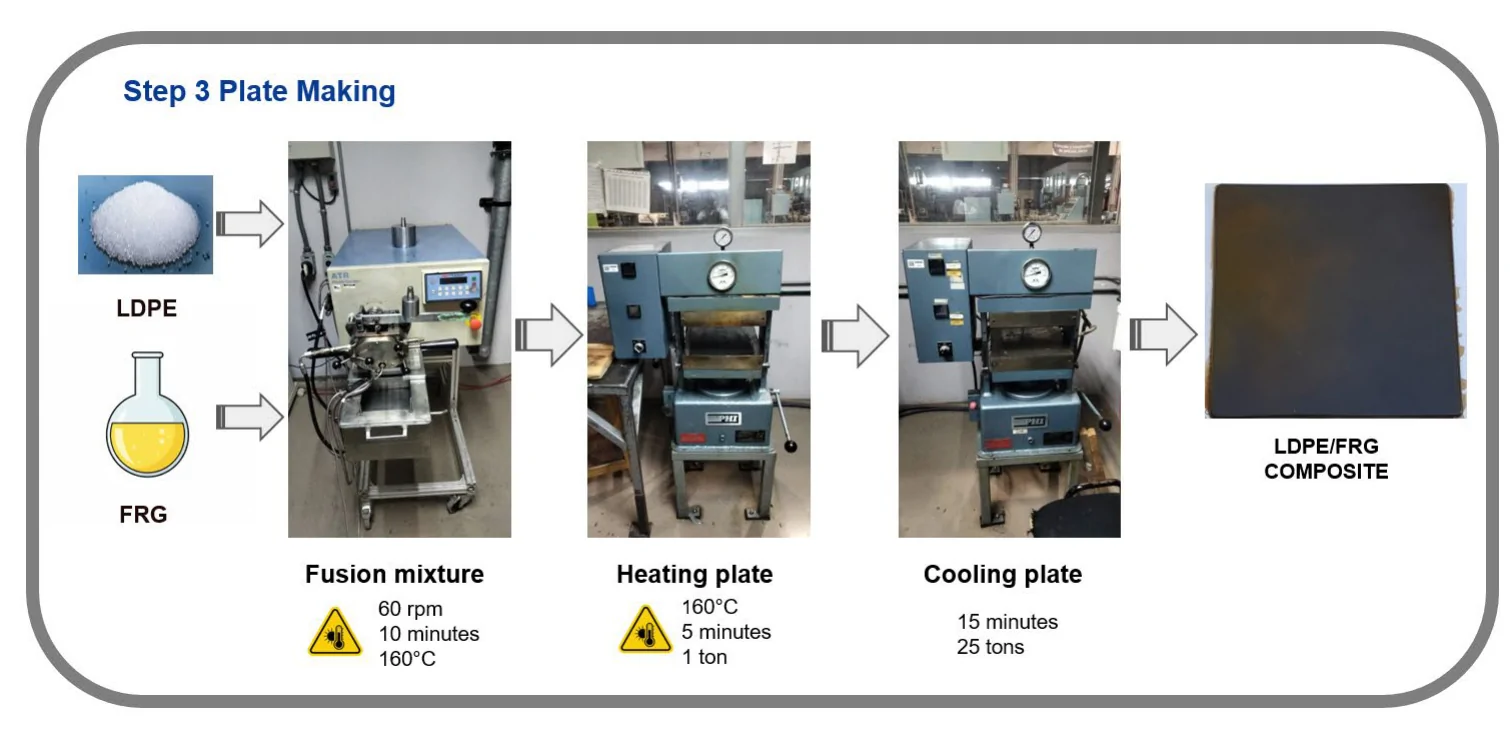
Schematic representation of the fabrication of LDPE/FRG composites through melt compounding followed by compression molding.

LDPE/FRG composites were prepared by melt compounding using a laboratory internal mixer (Brabender type). The mixing chamber was operated at 160 °C with a roller rotor speed of 60 rpm for 10 min. Neat LDPE was first introduced into the mixing chamber and allowed to reach a stable molten state before the incorporation of the FRG powder at a total loading of 20 wt%. Neat LDPE was processed under identical conditions and used as the reference material. Following melt compounding, the molten composites were compression molded using a PHI hydraulic press to produce plates for thermal, mechanical, and flammability characterization (Figure 3). The molding cycle consisted of preheating at 160 °C under a pressure of 1 ton for 5 min, followed by cooling under a pressure of 25 tons for 15 min using a water-cooled platen. The resulting plates were used to prepare specimens for UL-94 horizontal burning (ASTM D635 [18]), cone calorimetry, and the remaining characterization techniques. The compositions and sample designations are summarized in Table 1.

### 2.3. Characterization and Evaluations

#### 2.3.1. Structural, Morphological, and Particle Characterization

The chemical structure of the raw materials, extracted cellulosic scaffolds, and synthesized FRG powders was characterized by Fourier-transform infrared spectroscopy (FTIR) using a Thermo Scientific Nicolet iS10 spectrometer (Thermo Fisher Scientific, Madison, WI, USA) equipped with an attenuated total reflectance (ATR) diamond crystal. Spectra were recorded in the range of 4000–625 cm^−1^ with a spectral resolution of 4 cm^−1^ using 32 scans per spectrum. The crystalline structure of the samples was analyzed by X-ray diffraction (XRD) using a Siemens D500 diffractometer (Siemens AG, Karlsruhe, Germany). with Cu Kα radiation (*λ* = 1.5406 Å), operated at 35 kV and 25 mA. Diffractograms were collected over a *2θ* range of 10–80° using a step size of 0.07°. The morphology of the synthesized SiO_2_ particles, commercial nanocelluloses (CNC and CNF), and combustion residues was examined by high-resolution transmission electron microscopy (HR-TEM) using a FEI Titan 80–300 microscope (FEI Company, Eindhoven, The Netherlands) operated at 300 kV. The hydrodynamic particle size and colloidal stability of the synthesized SiO_2_ particles were determined by dynamic light scattering (DLS) using a Zetasizer Nano ZS90 (Malvern Instruments Ltd., Malvern, UK) at 25 °C. The textural properties of the synthesized SiO_2_ particles were evaluated by nitrogen adsorption–desorption measurements using an Autosorb iQ gas sorption analyzer (Quantachrome Instruments, Boynton Beach, FL, USA). Specific surface area was calculated using the Brunauer–Emmett–Teller (BET) method, while pore size distribution and pore volume were determined using the Barrett–Joyner–Halenda (BJH) method from the adsorption branch of the isotherm. Post-combustion residues were additionally examined by FTIR and scanning electron microscopy coupled with energy-dispersive X-ray spectroscopy (SEM/EDS) to assess their functional groups, surface morphology, and elemental composition in the analyzed regions.

#### 2.3.2. Thermal and Fire Performance Characterization

Thermogravimetric analysis (TGA) was performed using a TA Instruments Q500 thermogravimetric analyzer (TA Instruments, New Castle, DE, USA). Approximately 10 mg of sample was heated from 30 to 600 °C at 10 °C min^−1^ under a nitrogen atmosphere (50 mL min^−1^). At 600 °C, the atmosphere was switched to oxygen at the same flow rate, and the temperature was increased to 700 °C at 10 °C min^−1^ to determine the oxidative stability of the residual char. The thermal transitions of LDPE and the corresponding FRG composites were analyzed by differential scanning calorimetry (DSC) using a TA Instruments Q2000 calorimeter (TA Instruments, New Castle, DE, USA). Samples of approximately 8–10 mg were heated under nitrogen at a heating rate of 10 °C min^−1^. The melting temperature (*T_m_*), crystallization temperature (*T_c_*), melting enthalpy (*ΔH_m_*), and degree of crystallinity *(X_c_*) were determined from the thermograms. The flame-retardant performance of the composites was evaluated using the following standardized methods:

- UL-94 Horizontal Burning Test: Horizontal burning behavior was evaluated according to the UL 94 standard using specimens measuring 125 × 13 × 3 mm^3^. The burning rate was calculated from the time required for the flame front to travel between the reference marks.
- Cone Calorimetry: Cone calorimeter tests were conducted according to ASTM E1354 [19] using specimens measuring 100 × 100 × 3 mm^3^ under an external heat flux of 35 kW m^−2^. Heat release rate (*HRR*), peak heat release rate (*pHRR*), total heat release (*THR*), time to ignition (*TTI*), and total smoke production (*TSP*) were recorded.

#### 2.3.3. Mechanical Characterization

Tensile properties were determined according to ASTM D638 [20] using Type IV dog-bone specimens. Tests were performed at a crosshead speed of 5 mm min^−1^ using an Instron universal testing machine equipped with a 25 mm gauge-length extensometer. Tensile strength and elongation at break were obtained from the corresponding stress–strain curves. Flexural properties were evaluated according to ASTM D790 [21] using a three-point bending configuration on an Instron 4301 universal testing machine equipped with a 500 N load cell. Prior to testing, all specimens were conditioned at 23 ± 2 °C and 50 ± 5% relative humidity for 48 h. At least five specimens were tested for each formulation, and the results are reported as mean values ± standard deviation.

## 3. Results and Discussion

The FRG consists of four functional components: a renewable cellulosic scaffold acting as the carbon source, APP as the acid source, ADA as the blowing agent, and amorphous SiO_2_ as the inorganic reinforcement secondary scaffold. After fabrication, the FRG powders were incorporated into LDPE through melt compounding and compression molding to produce the corresponding composite formulations for structural, thermal, mechanical, and fire-performance evaluation.

### 3.1. Characterization of Synthesized SiO_2_ Scaffolds

#### 3.1.1. FTIR and XRD Characterization

**Figure 4:**
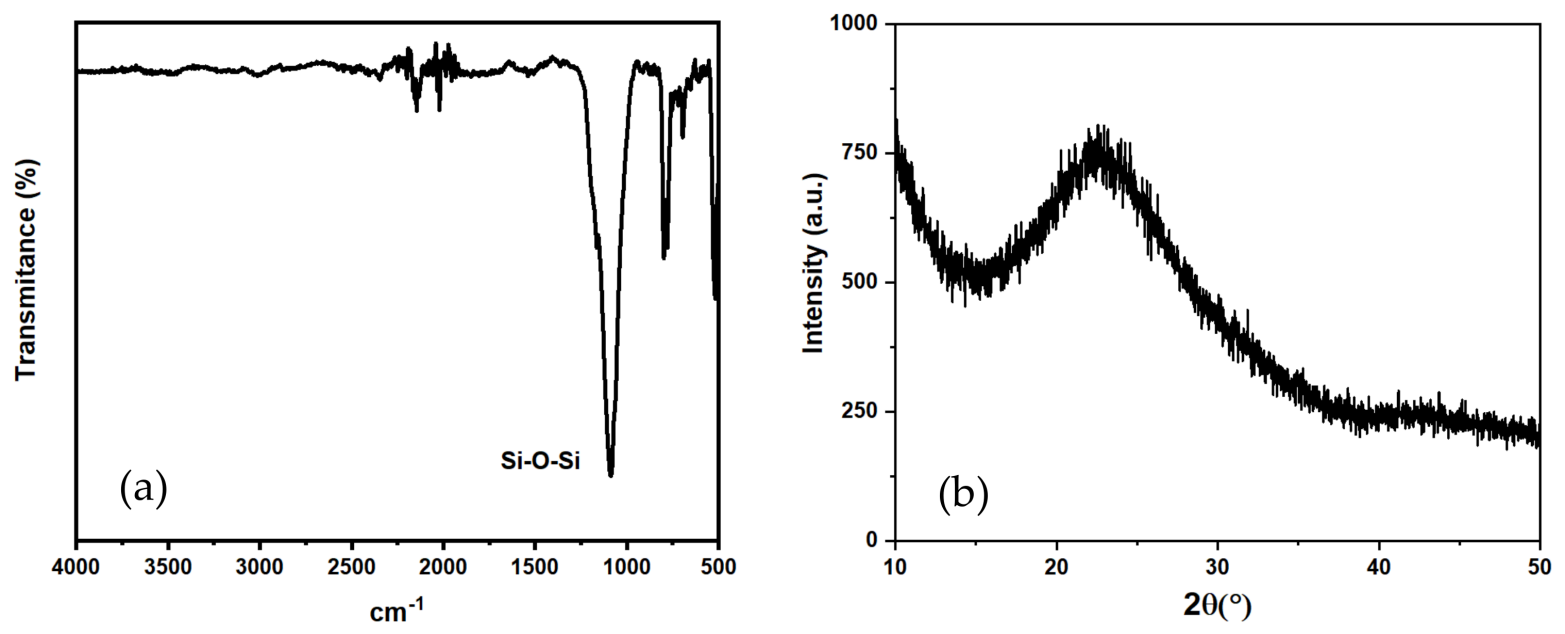
Structural and vibrational characterization of the synthesized sub-micron SiO_2_ spheres: (**a**) FTIR spectrum highlighting the characteristic Si–O–Si siloxane network, and (**b**) XRD diffractogram.

The chemical structure and phase composition of the synthesized submicron SiO_2_ particles were characterized by FTIR and X-ray diffraction. The FTIR spectrum (Figure 4a) exhibits the characteristic vibrational bands of amorphous silica. The intense absorption band centered at approximately 1080 cm^−1^ is assigned to the asymmetric stretching vibration of Si–O–Si bonds, whereas the bands located at 800 cm^−1^ and 460 cm^−1^ correspond to the symmetric stretching and bending vibrations of the siloxane network, respectively [22]. These absorption bands are characteristic of amorphous silica synthesized by sol–gel methods. No significant absorption band was observed in the 3200–3600 cm^−1^ region, where the stretching vibration of hydroxyl groups (Si–OH) and adsorbed water is typically detected. This result suggests that the calcination treatment at 600 °C effectively reduced the concentration of surface hydroxyl groups, yielding a silica surface with lower polarity. Such surface characteristics may contribute to improved compatibility with hydrophobic polymer matrices during composite processing. The crystalline structure of the synthesized particles was further evaluated by X-ray diffraction (Figure 4b). The diffractogram exhibits a broad diffuse halo centered at approximately 2θ = 24°, extending from approximately 15° to 30°, which is characteristic of amorphous silica lacking long-range crystalline order [23]. No diffraction peaks associated with crystalline silica polymorphs, including quartz or cristobalite, were detected, indicating that the synthesized material is predominantly amorphous.

#### 3.1.2. Nitrogen Adsorption–Desorption Analysis

**Figure 5:**
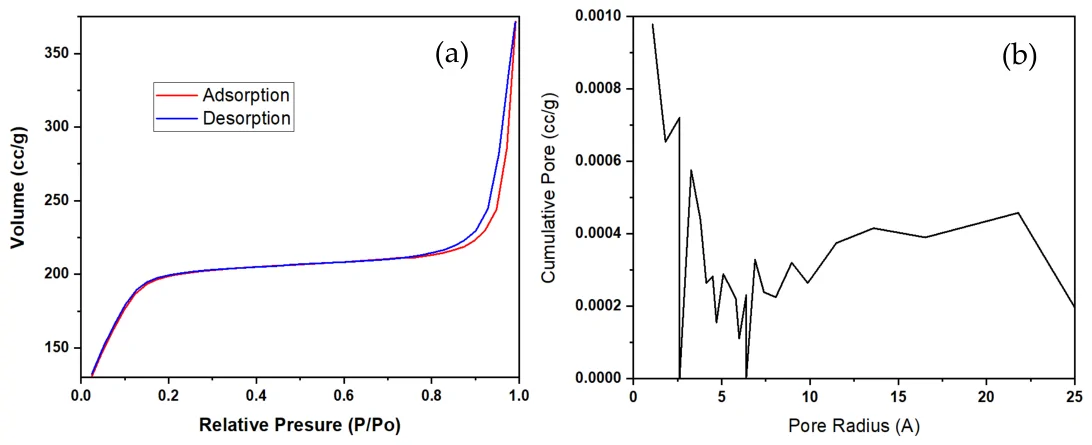
Nitrogen adsorption–desorption isotherm of the synthesized SiO_2_ particles (**a**) measured at 77 K and (**b**) BJH pore size distribution.

The textural properties of the synthesized SiO_2_ particles were evaluated by nitrogen adsorption–desorption measurements. As shown in Figure 5a, the adsorption–desorption isotherm exhibits a type IV profile with a pronounced hysteresis loop at high relative pressures, which is characteristic of mesoporous materials according to the IUPAC classification. This behavior indicates the formation of a porous silica structure following calcination and removal of the CTAB template. BET analysis yielded a specific surface area of 698.46 m^2^ g^−1^, a BET constant C of 878.48, and a correlation coefficient (*R^2^*) of 0.9971, indicating an excellent fit of the experimental data to the BET model. BJH analysis revealed a total pore volume of 0.273 cm^3^ g^−1^ and a dominant pore diameter centered at approximately 9–10 nm, which lies within the mesoporous range defined by IUPAC (2–50 nm). The combination of the type IV isotherm, hysteresis loop, high specific surface area, and BJH pore size distribution confirms the mesoporous nature of the synthesized silica. Such characteristics are desirable for the proposed FRG system because the large accessible surface area may facilitate interactions among the SiO_2_, APP, ADA, and the lignocellulosic materials, potentially influencing the formation and stability of the protective residue generated during combustion.

#### 3.1.3. Morphological Analysis and Particle Size Distribution

**Figure 6:**
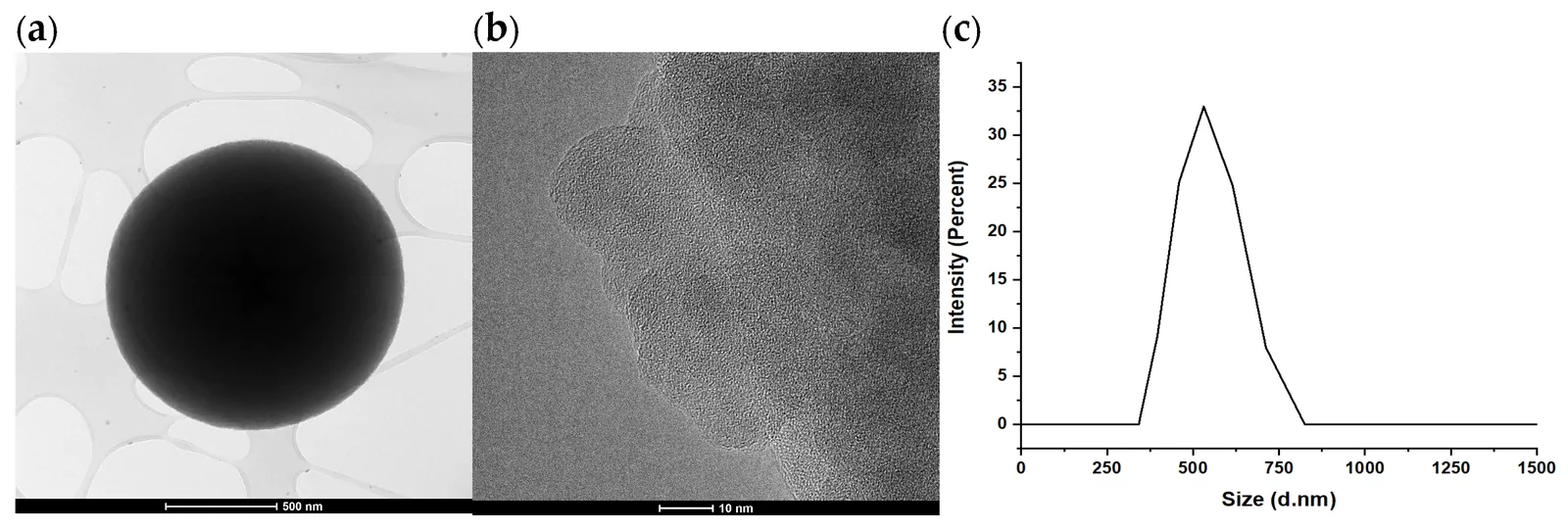
Morphological and particle size distribution analysis of the synthesized SiO_2_: low-magnification TEM micrograph (**a**), high-resolution TEM (**b**), and DLS particle size distribution (**c**).

The morphology and particle size distribution of the synthesized SiO_2_ particles were investigated by transmission electron microscopy (TEM) and dynamic light scattering (DLS). The TEM image shown in Figure 6a reveals a predominantly spherical morphology with relatively uniform particle dimensions and limited particle agglomeration. The particles exhibit well-defined boundaries, indicating the formation of discrete silica spheres during the synthesis process. The high-resolution TEM micrograph (Figure 6b) shows a homogeneous internal contrast without visible lattice fringes, which is consistent with the amorphous structure previously identified by XRD analysis. No evidence of crystalline domains was observed within the analyzed particles. The particle size distribution obtained by DLS (Figure 6c) exhibits a narrow monomodal profile with an average hydrodynamic diameter of approximately 533 nm, confirming the formation of submicron silica particles with relatively uniform size distribution [24]. The combination of spherical morphology, submicron particle size, and high surface area is advantageous for incorporation into the proposed FRG system. These characteristics may facilitate particle dispersion within the hybrid architecture and contribute to the development of thermally stable protective residues during combustion [7].

### 3.2. Structural, Crystalline, and Thermal Characterization of Cellulose

#### 3.2.1. FTIR Analysis of Waste-Derived and Commercial Cellulose

**Figure 7:**
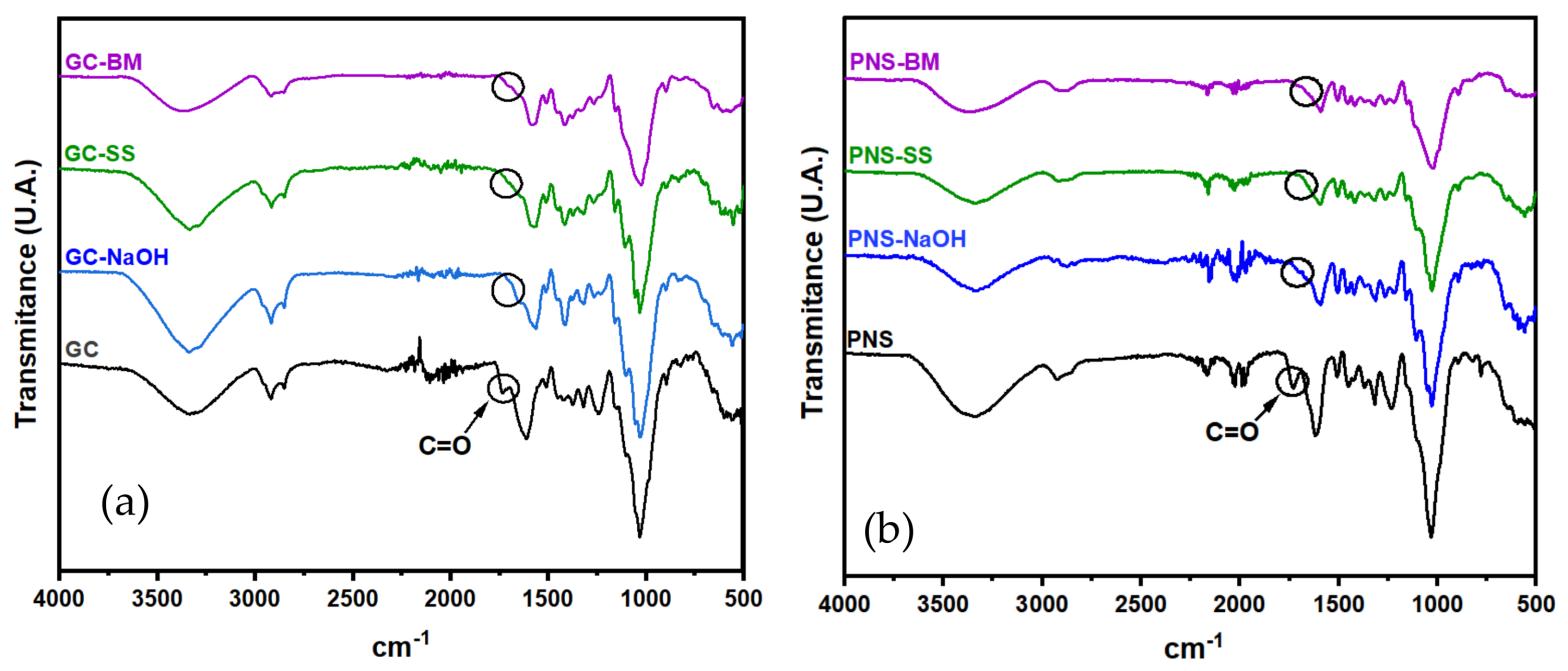
FTIR spectra of cellulose derived from (**a**) GC and (**b**) PNS following alkaline (NaOH), steam explosion (SS), and ball-milling (BM) treatments.

**Figure 8:**
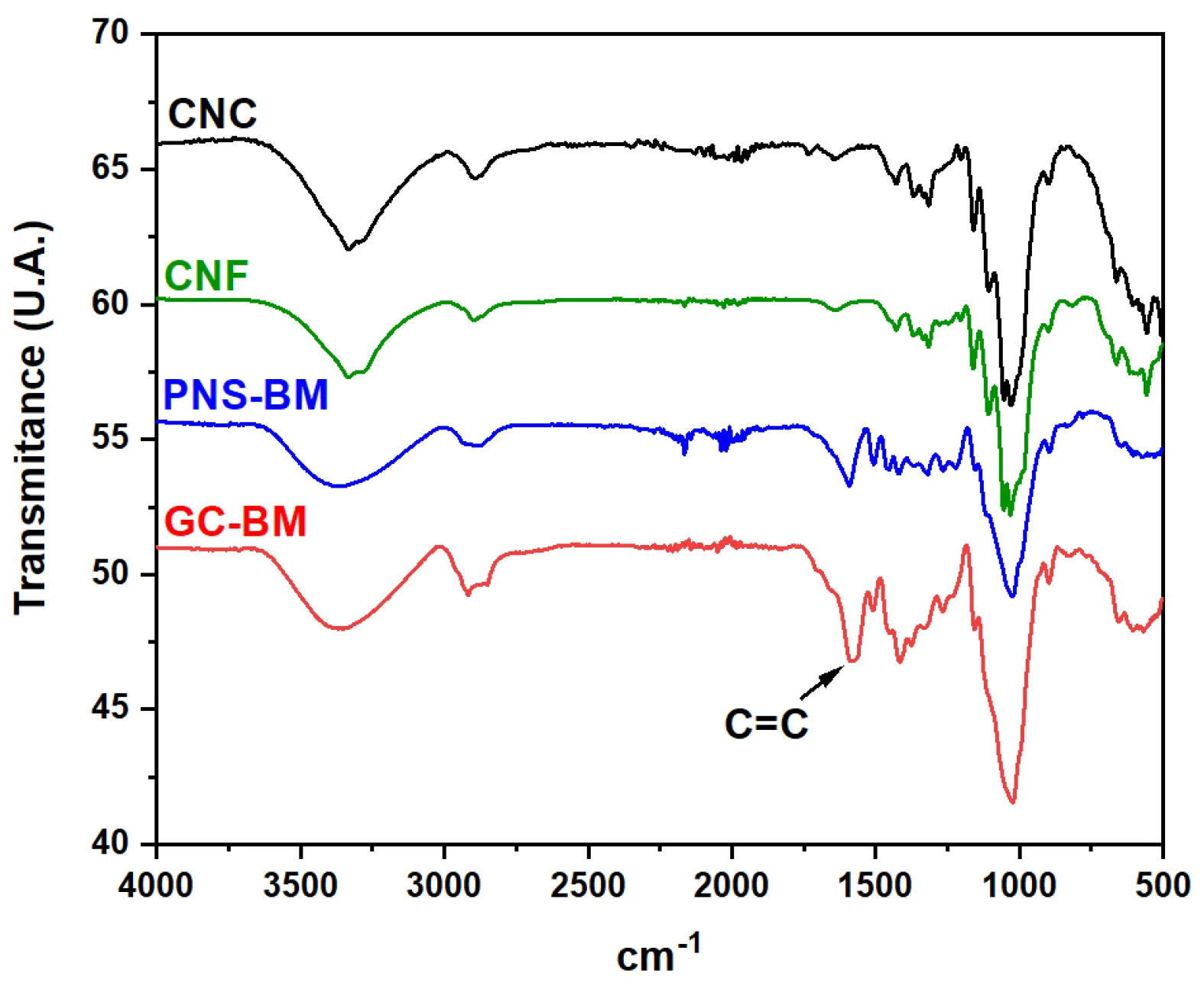
FTIR spectral comparison of commercial nanocelluloses (CNC, CNF) and agricultural waste celluloses (PNS, GC).

The chemical structure of the extracted agricultural cellulose (PNS and GC) was characterized by Fourier-transform infrared spectroscopy (FTIR) and compared with commercial cellulose nanocrystals (CNC) and cellulose nanofibrils (CNF). The spectra presented in Figure 7 and Figure 8 exhibit the characteristic absorption bands of cellulose in all four samples. A broad absorption band centered at approximately 3340 cm^−1^ is assigned to the stretching vibration of hydroxyl groups involved in intra- and intermolecular hydrogen bonding, while the band at approximately 2900 cm^−1^ corresponds to the stretching vibration of aliphatic C–H groups. The fingerprint region further confirms the preservation of the cellulose backbone through the characteristic absorption bands at approximately 1160 cm^−1^, assigned to the asymmetric stretching vibration of the β-(1→4)-glycosidic C–O–C linkage, and at 1030 cm^−1^, corresponding to C–O stretching within the pyranose ring. These bands were observed in all four samples, indicating that the cellulose structure was preserved after the extraction treatment. Distinct differences were observed between the agricultural cellulosic scaffolds and the commercial nanocelluloses. The spectra of PNS and GC exhibit absorption bands centered at approximately 1604 cm^−1^ and 1510 cm^−1^, which are attributed to the C=C aromatic vibrations of lignin. In addition, a shoulder near 1240 cm^−1^, assigned to C–O stretching in lignin, was detected in both waste-derived samples [25]. These lignin-associated bands were absent or considerably less intense in the commercial CNC and CNF samples, consistent with industrial purification and bleaching, but not providing a quantitative measure of lignin content. A weak absorption band near 1735 cm^−1^ was observed in the PNS sample and is attributed to the C=O stretching vibration of residual acetyl or ester groups associated with minor amounts of hemicellulose remaining after the alkaline treatment. This band was considerably less evident in the remaining samples, suggesting a more extensive removal of hemicellulosic components during processing [26]. Overall, the FTIR results demonstrate that the extraction procedure successfully preserved the cellulose backbone while retaining lignin-associated functional groups in the waste-derived cellulosic scaffolds. In contrast, the commercial CNC and CNF exhibited the characteristic spectral features of highly purified cellulose. These compositional differences provide the chemical basis for the subsequent evaluation of their influence on thermal degradation, char formation, and flame-retardant performance using complementary thermal and combustion analyses. Although lignin is reported to promote char formation, FTIR spectroscopy provides qualitative rather than quantitative data; therefore, these absorption bands cannot be used to establish a proportional relationship with residue yield.

#### 3.2.2. X-Ray Diffraction Analysis of Waste-Derived and Commercial Cellulosic Scaffolds

**Figure 9:**
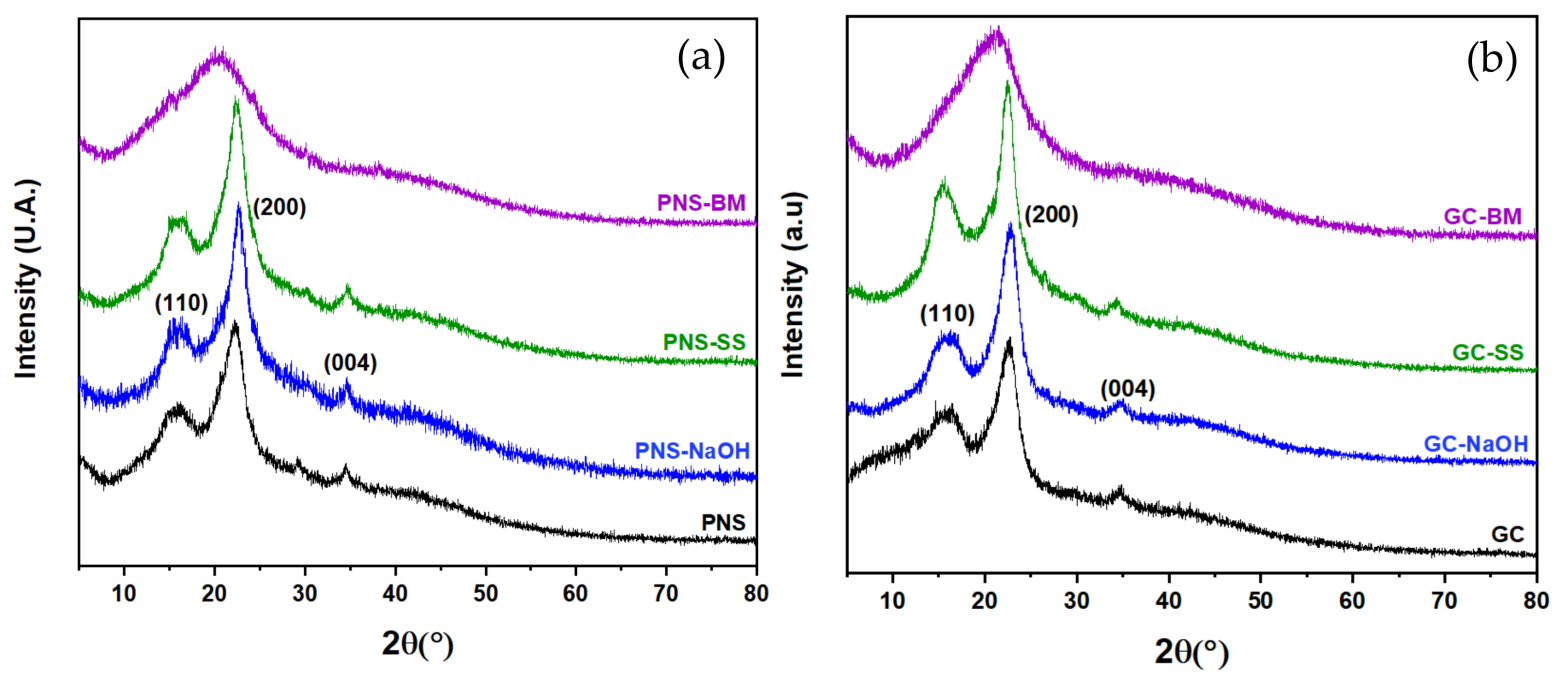
XRD patterns illustrating the structural evolution of cellulose extracted from (**a**) PNS and (**b**) GC via alkaline treatment (NaOH), steam explosion (SS), and ball-milling (BM).

**Figure 10:**
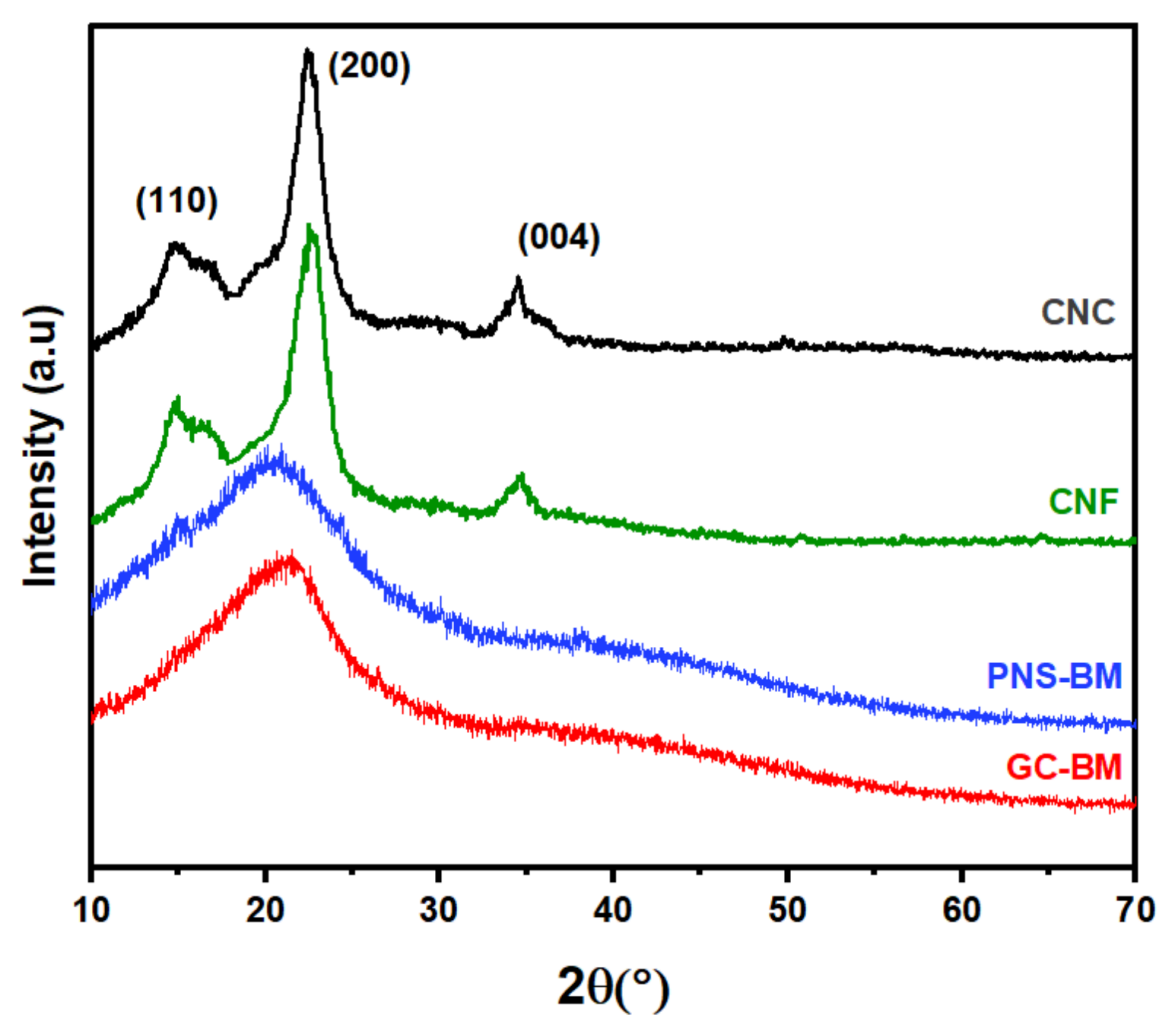
Comparative characterization of pure cellulose matrices: Nanocrystalline Cellulose (CNC), Nanofibrillated Cellulose (CNF), treated pecan nutshell cellulose (PNS-BM), and Guayule Cellulose (GC-BM).

The evolution of the crystalline structure during cellulose extraction from pecan nutshell (PNS) and guayule bagasse (GC) was monitored by X-ray diffraction (XRD). The diffraction patterns of the raw biomass (Figure 9) exhibit the characteristic reflections of native cellulose I, including the principal diffraction peak at approximately *2θ* = 22.5°, assigned to the (200) crystallographic plane, together with the reflections located near 15° and 16.5°, corresponding to the (1–10) and (110) planes, respectively. The relatively broad diffraction peaks observed in the untreated biomass are attributed to the presence of amorphous constituents, mainly lignin and hemicellulose, which contribute to the diffuse background and reduce the apparent crystalline order of the native lignocellulosic material. Following the alkaline treatment and steam explosion, the diffraction peak corresponding to the (200) plane became more intense and better defined for both PNS and GC. This behavior is consistent with the partial removal of amorphous components, resulting in an increase in the relative crystallinity of the remaining cellulose fraction. The subsequent ball-milling treatment produced a moderate broadening of the diffraction peaks together with a slight reduction in peak intensity. These changes are commonly associated with a decrease in crystallite size and partial disruption of the ordered cellulose domains induced by mechanical processing. Nevertheless, the characteristic diffraction pattern of cellulose I remained unchanged throughout the extraction process, indicating that the applied treatment modified the degree of structural order without altering the native cellulose polymorph. The crystalline structures of the final cellulosic scaffolds are compared in Figure 10. All samples exhibit the characteristic diffraction pattern of cellulose I, with the principal (200) reflection located between *2θ* = 22.36° and 22.60°, confirming that the native crystalline structure was preserved after extraction and processing [27,28].

Commercial cellulose nanocrystals (CNC) and cellulose nanofibrils (CNF) display narrower and more intense diffraction peaks than the waste-derived PNS and GC scaffolds, reflecting their higher degree of crystalline order as a consequence of industrial purification and controlled nanocellulose production. In contrast, the diffraction peaks of PNS and GC are slightly broader, which is attributed to the combined effects of mechanical ball milling and the retention of residual amorphous constituents within the extracted fibers. Importantly, no diffraction peaks characteristic of cellulose II were detected in any of the extracted samples, indicating that the alkaline treatment and steam explosion preserved the native cellulose I polymorph without inducing a significant crystalline phase transformation. Overall, the XRD results demonstrate that the extraction process effectively enriched the cellulose fraction while maintaining its native crystalline structure. The observed differences among the commercial and waste-derived cellulosic scaffolds are primarily associated with variations in crystalline order rather than changes in cellulose polymorphism. These structural characteristics provide a basis for interpreting the thermal degradation and combustion behavior discussed in the following sections, where the influence of cellulose crystallinity and residual lignin on char formation and flame-retardant performance is evaluated using complementary thermal and fire characterization techniques [29].

#### 3.2.3. Thermogravimetric Analysis (TGA) and Derivative Thermogravimetry (DTG)

**Figure 11:**
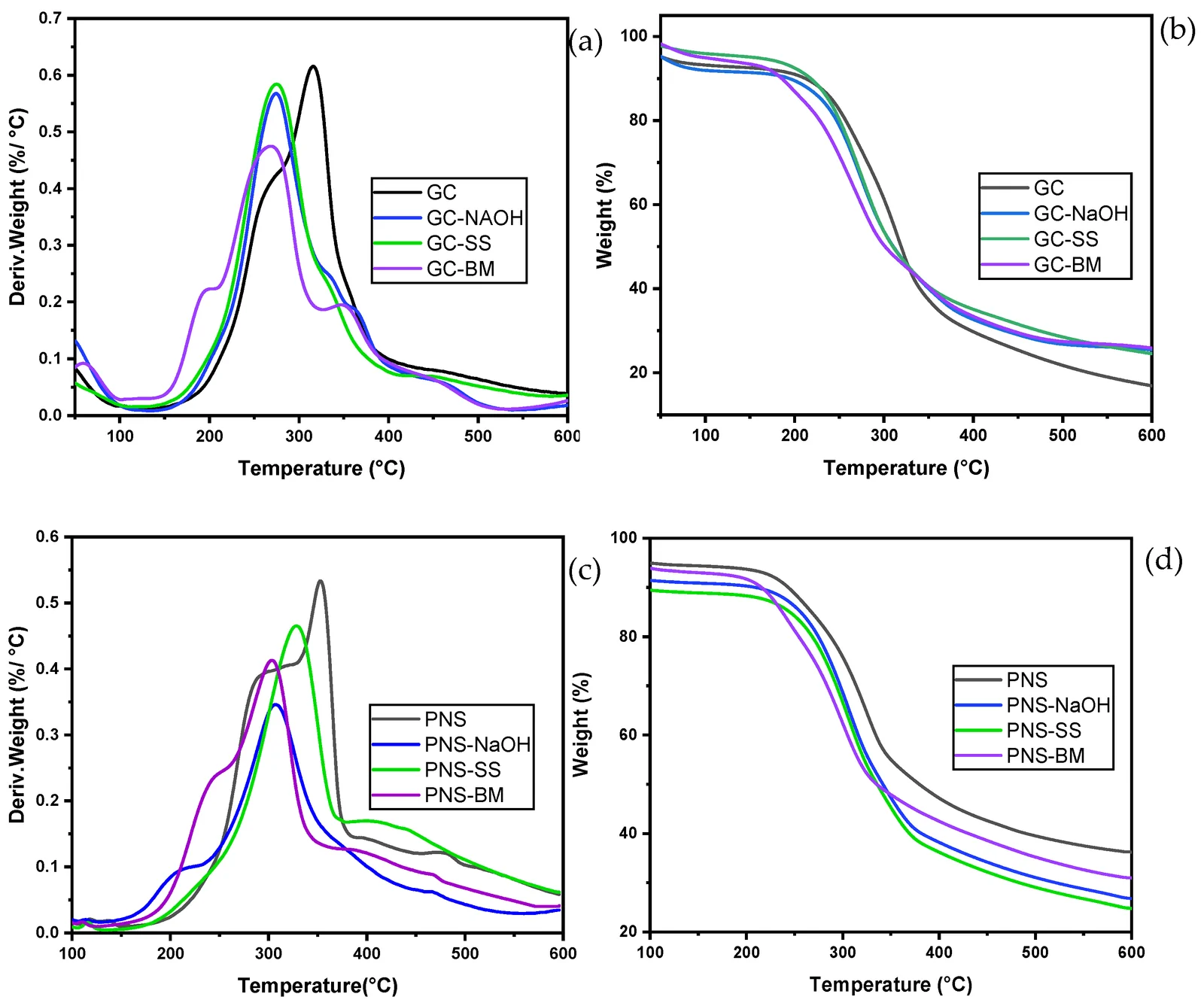
DTG and TGA curves depicting the thermal degradation of cellulose extracted from GC (**a**,**b**) and PNS (**c**,**d**) throughout the multistep process.

The thermal degradation behavior of the cellulosic materials throughout the extraction process was evaluated by thermogravimetric analysis (TGA) and derivative thermogravimetry (DTG). The thermograms shown in Figure 11a,b reveal the characteristic multistep degradation profile of the guayule cellulose (GC), and Figure 11c,d shows the pecan nutshell (PNS) biomass. An initial mass loss below 100 °C is attributed to the evaporation of physically adsorbed moisture. A second degradation stage between approximately 200 and 300 °C is associated with the decomposition of hemicellulose and other low-molecular-weight amorphous constituents. The principal degradation event, occurring between approximately 300 and 380 °C, corresponds predominantly to the thermal decomposition of cellulose [26,27]. Following the alkaline treatment and steam explosion, the DTG curves exhibit a progressive reduction in the degradation shoulder assigned to hemicellulose together with a displacement of the maximum degradation temperature toward higher temperatures. This behavior is consistent with the partial removal of amorphous constituents during the extraction process, resulting in an enrichment of the cellulose fraction and an increase in its relative thermal stability. In contrast, the subsequent ball-milling treatment produced a slight decrease in temperature decomposition accompanied by broader degradation peaks. This behavior is attributed to the reduction in crystallite size and the partial disruption of the ordered cellulose domains, in agreement with the structural changes observed by XRD analysis [30].

**Figure 12:**
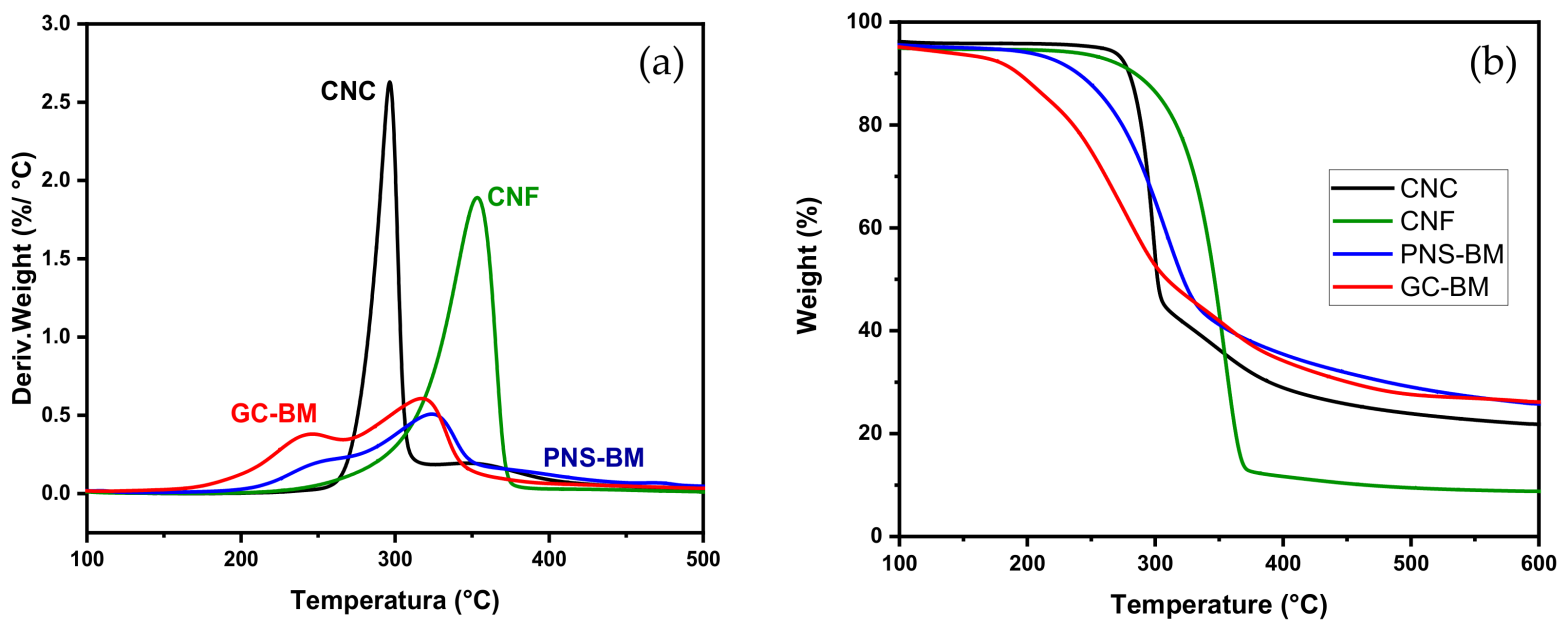
Comparative TGA (**a**) and DTG (**b**) thermograms of commercial cellulose nanocrystals (CNC), cellulose nanofibrils (CNF), and waste-derived cellulosic scaffolds obtained from pecan nutshell (PNS) and guayule bagasse (GC) under a nitrogen atmosphere.

Figure 12 compares the thermal degradation behavior of the final cellulosic scaffolds with that of commercial CNC and CNF. Although all four materials retain the characteristic cellulose I crystalline structure, significant differences in their thermal decomposition profiles are observed as a consequence of their origin and processing history. Commercial cellulose nanocrystals (CNC) exhibit the lowest thermal stability, with the maximum degradation rate occurring near 300 °C. This behavior has been widely reported for sulfuric acid-hydrolyzed cellulose nanocrystals and is commonly attributed to the presence of sulfate ester groups introduced during acid hydrolysis, which catalyze cellulose degradation [31]. Cellulose nanofibrils (CNF) exhibit a higher decomposition temperature than CNC, indicating improved thermal stability. Unlike acid-hydrolyzed nanocrystals, mechanically fibrillated cellulose contains fewer thermally unstable surface groups, delaying the onset of thermal degradation. The waste-derived cellulosic scaffolds (PNS and GC) exhibit broader degradation profiles than the commercial nanocelluloses. In particular, GC presents a wider degradation interval beginning at lower temperatures, which is consistent with the lower structural order observed in the XRD analysis. Similarly, PNS exhibits a broad degradation profile but retains a larger residual mass at the end of the thermal analysis. Residual lignin and other aromatic constituents remaining after extraction may contribute to this behavior because lignin decomposes over a broader temperature range than cellulose [32]. The combined XRD and TGA results indicate that the extraction procedure successfully preserved the native cellulose I structure while maintaining compositional differences between the waste-derived and commercial cellulosic scaffolds. In particular, the higher residual mass observed for PNS reflects its greater carbonization potential during thermal degradation. However, the extent to which this enhanced carbon yield contributes to the formation of a protective char layer and improves the flame-retardant performance of the Hybrid Flame-Retardant Granade (FRG) is evaluated in the subsequent fire-performance analyses.

#### 3.2.4. Particle Size

**Figure 13:**
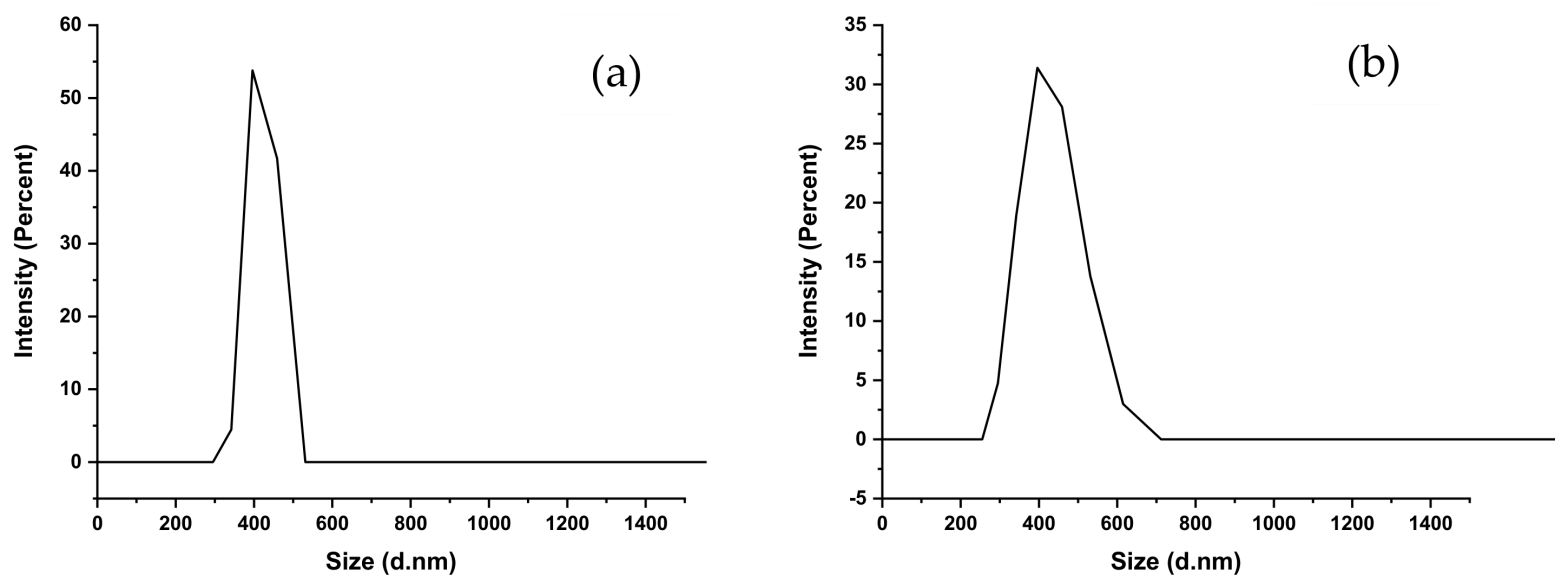
DLS particle size distribution of (**a**) GC-BM and (**b**) PNS-BM.

The particle size distributions of PNS-BM and GC-BM were evaluated via Dynamic Light Scattering (DLS), as shown in Figure 13, whereas the dimensions for the commercial nanocelluloses were referenced from the supplier’s technical data. GC-BM exhibits a hydrodynamic diameter range of 295–531 nm, with an average size of 396 nm. Similarly, PNS-BM displays an identical average size of 396 nm, albeit with a broader distribution ranging from 255 to 712 nm. Although both extracted celluloses achieved the same average dimension, the wider size interval observed for PNS is attributed to the inherent morphological-chemical nature of its agricultural precursor. Nonetheless, these results highlight the critical role of the mechanical ball-milling process in effectively reducing the particles to the observed submicron scale. For reference, the commercial CNC is specified to have a diameter of 2.3–4.5 nm and a length of 44–108 nm, whereas CNF features a diameter of 20–70 nm with variable micrometer-scale lengths.

### 3.3. Flame-Retardant Properties

#### 3.3.1. Thermogravimetric Analysis of the Hybrid Flame-Retardant Grenade (FRG) Systems

**Figure 14:**
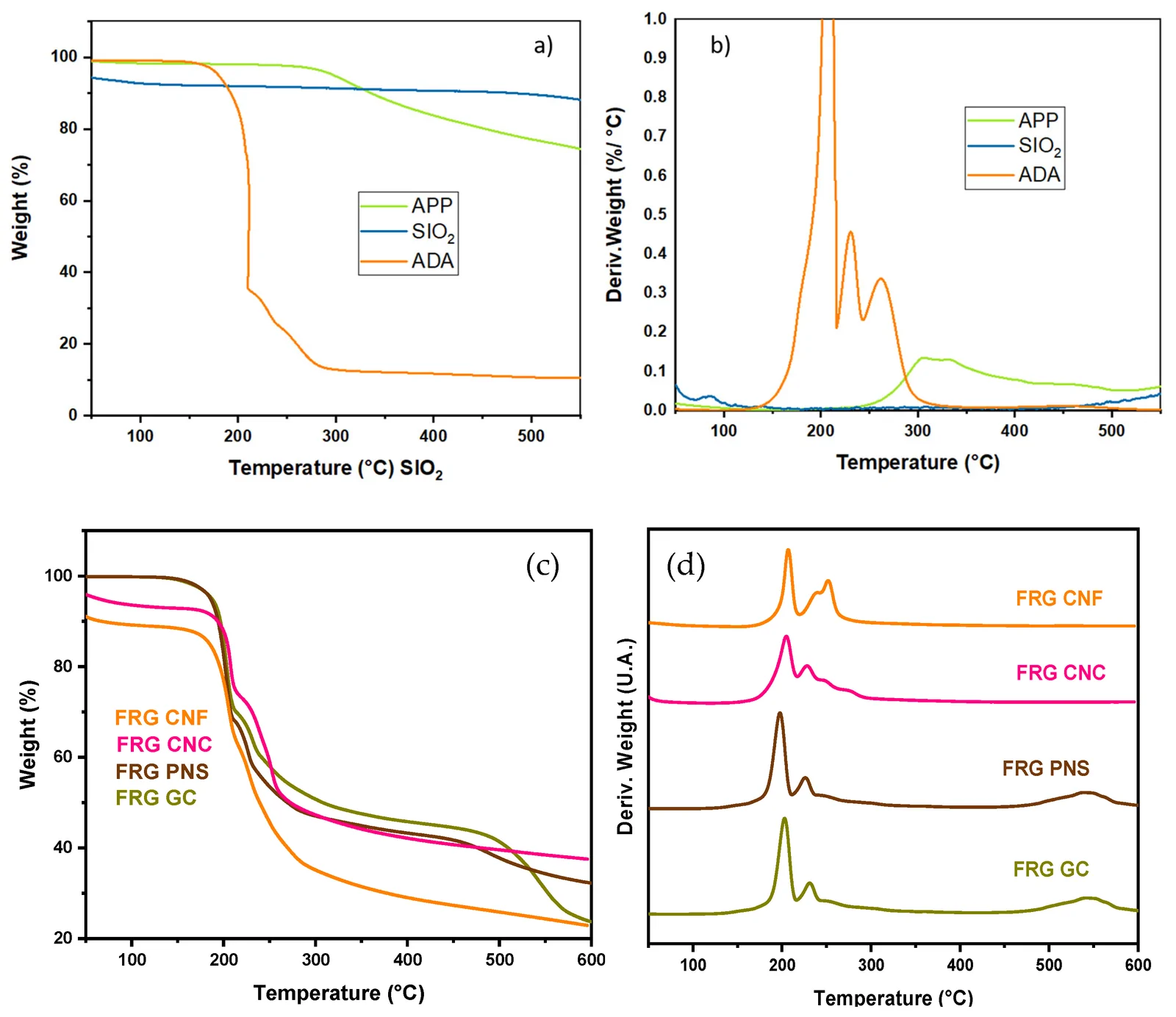
(**a**) TGA thermograms of the individual flame-retardant components (APP, SiO_2_, and ADA); (**b**) corresponding DTG curves; (**c**) TGA thermograms of the FRG systems prepared with different cellulosic scaffolds (FRG-CNF, FRG-CNC, FRG-PNS, and FRG-GC); and (**d**) corresponding DTG curves illustrating the thermal degradation behavior of the assembled hybrid architectures.

The thermal degradation behavior of the individual flame-retardant components, including APP, SiO_2_, and ADA, together with the corresponding cellulose to obtain FRG systems, using cellulose nanofibrils (FRG-CNF), cellulose nanocrystals (FRG-CNC), pecan nutshell cellulose (FRG-PNS), and guayule cellulose (FRG-GC), was evaluated by thermogravimetric analysis (TGA) and derivative thermogravimetry (DTG). This analysis was performed to examine the thermal decomposition behavior of the individual constituents and to determine how their assembly within the FRG architecture modifies the overall degradation profile. As shown in Figure 14a,b, the synthesized amorphous SiO_2_ exhibited negligible mass loss throughout the investigated temperature range as expected, confirming its high thermal stability under the experimental conditions. In contrast, APP initiated progressive thermal decomposition above approximately 300 °C, whereas ADA exhibited a major degradation event centered near 200 °C, corresponding to the evolution of gaseous decomposition products, followed by a second, less pronounced mass-loss stage at higher temperatures. These thermal profiles agree well with the decomposition behavior commonly reported for these flame-retardant components [33,34]. The thermal behavior of the assembled FRG systems differs substantially from that of the isolated constituents (Figure 14c,d). Rather than exhibiting independent degradation events associated with APP, ADA, and the cellulosic scaffold, the hybrid architectures display broader and partially overlapping decomposition stages, indicating thermal interactions among the individual components during pyrolysis. This behavior reflects the integrated nature of the FRG architecture, in which degradation processes occur over overlapping temperature intervals, thereby facilitating an intumescent flame-retardant mechanism. Among the investigated formulations, the FRG systems prepared from the waste-derived cellulosic scaffolds (FRG-PNS and FRG-GC) retained a similar fraction of residual mass (20–40 wt% at temperatures above approximately 400 °C to the corresponding systems based on commercial nanocelluloses (FRG-CNC and FRG-CNF). This behavior is consistent with the broader degradation profiles previously observed for the isolated PNS and GC cellulose and may reflect contributions from residual lignin and other constituents remaining after extraction, together with structural differences among the scaffolds (SiO_2_). Overall, the TGA and DTG results demonstrate that the incorporation of waste-derived cellulosic scaffolds modifies the thermal degradation behavior of the FRG systems and promotes similar residual char formation to that of the corresponding formulations prepared with commercial nanocelluloses. The implications of these differences for the morphology of the protective residue and the flame-retardant performance of the LDPE composites are discussed in the following sections through combustion testing.

#### 3.3.2. FTIR Spectroscopy: Chemical Integration Analysis

**Figure 15:**
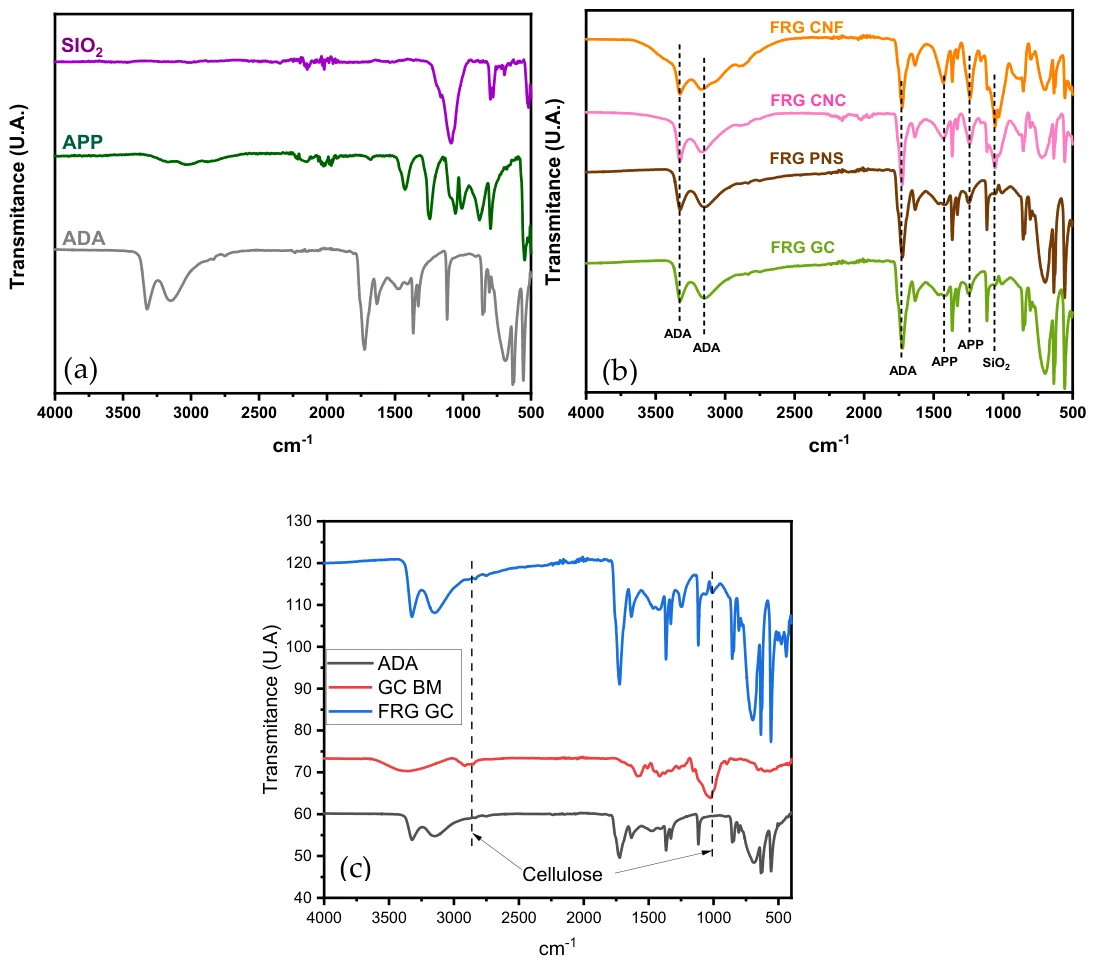
Comparative FTIR spectra of (**a**) individual components, (**b**) hybrid FRG systems formulated with various cellulosic sources, and (**c**) the organic constituent components of FRG.

The Fourier-transform infrared (FTIR) spectra of the individual components and formulated hybrid Flame-Retardant Grenade (FRG) systems are presented in Figure 15a–c. The spectra retain the characteristic absorption bands of the individual constituents while exhibiting subtle modifications associated with the assembly of the hybrid architectures. Compared with the isolated cellulosic scaffolds, all FRG formulations display a broader hydroxyl stretching band within the 3200–3500 cm^−1^ region accompanied by a slight shift toward lower wavenumbers. This spectral behavior is consistent with the establishment of intermolecular hydrogen-bonding interactions among the hydroxyl groups of cellulose, the surface silanol groups of the synthesized SiO_2_ particles, amide groups of ADA, and phosphate-containing species derived from ammonium polyphosphate (APP). The principal vibrational bands associated with cellulose, including the C–H stretching vibration near 2900 cm^−1^, the asymmetric C–O–C stretching of the β-glycosidic linkages around 1160 cm^−1^, and the C–O stretching vibrations of the pyranose ring near 1030 cm^−1^, remain clearly identifiable after incorporation into the hybrid architectures. Likewise, the characteristic Si–O–Si asymmetric stretching band centered near 1080 cm^−1^ confirms the successful incorporation of the synthesized amorphous silica into all FRG formulations. The formulations prepared from waste-derived cellulosic scaffolds (FRG-PNS and FRG-GC) exhibit additional absorption bands between 1510 and 1600 cm^−1^, corresponding to the aromatic skeletal vibrations characteristic of residual lignin. These bands are absent in the commercial nanocellulose-based formulations (FRG-CNC and FRG-CNF), confirming that the extraction procedure preserved part of the native lignin fraction in the agricultural residues. This observation agrees with the FTIR characterization previously presented for the isolated cellulosic materials. Overall, the FTIR spectra indicate that the characteristic functional groups of the individual constituents are preserved after assembly of the FRG systems. The high degree of spectral similarity among the different formulations further suggests that the ultrasound-assisted assembly procedure produces chemically comparable hybrid architectures regardless of the botanical origin of the cellulosic materials.

#### 3.3.3. X-Ray Diffraction (XRD): Structural Integrity and Crystallinity

**Figure 16:**
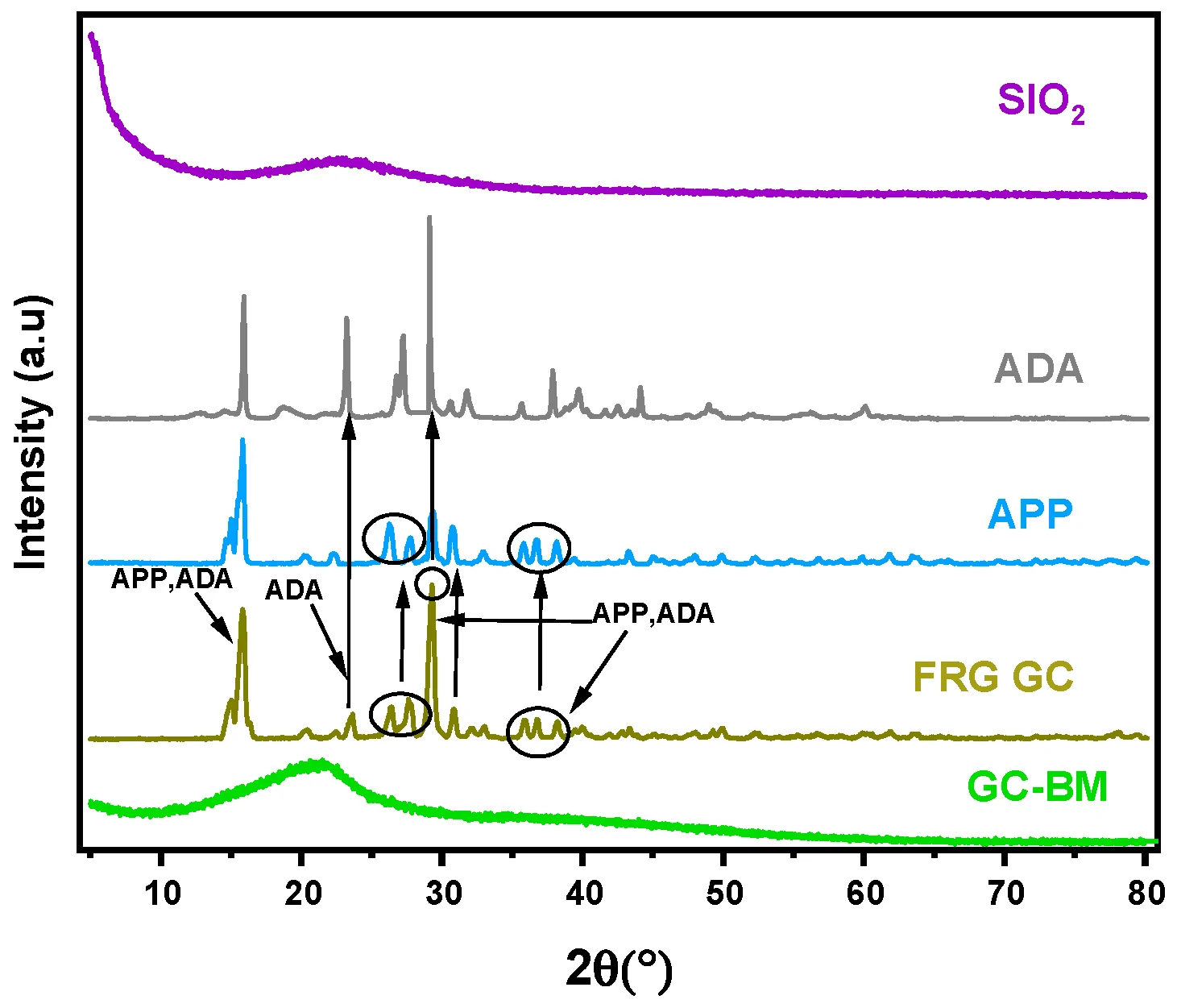
XRD patterns of the flame-retardant grenade of guayule (FRG GC) compared with those of its constituent components.

**Figure 17:**
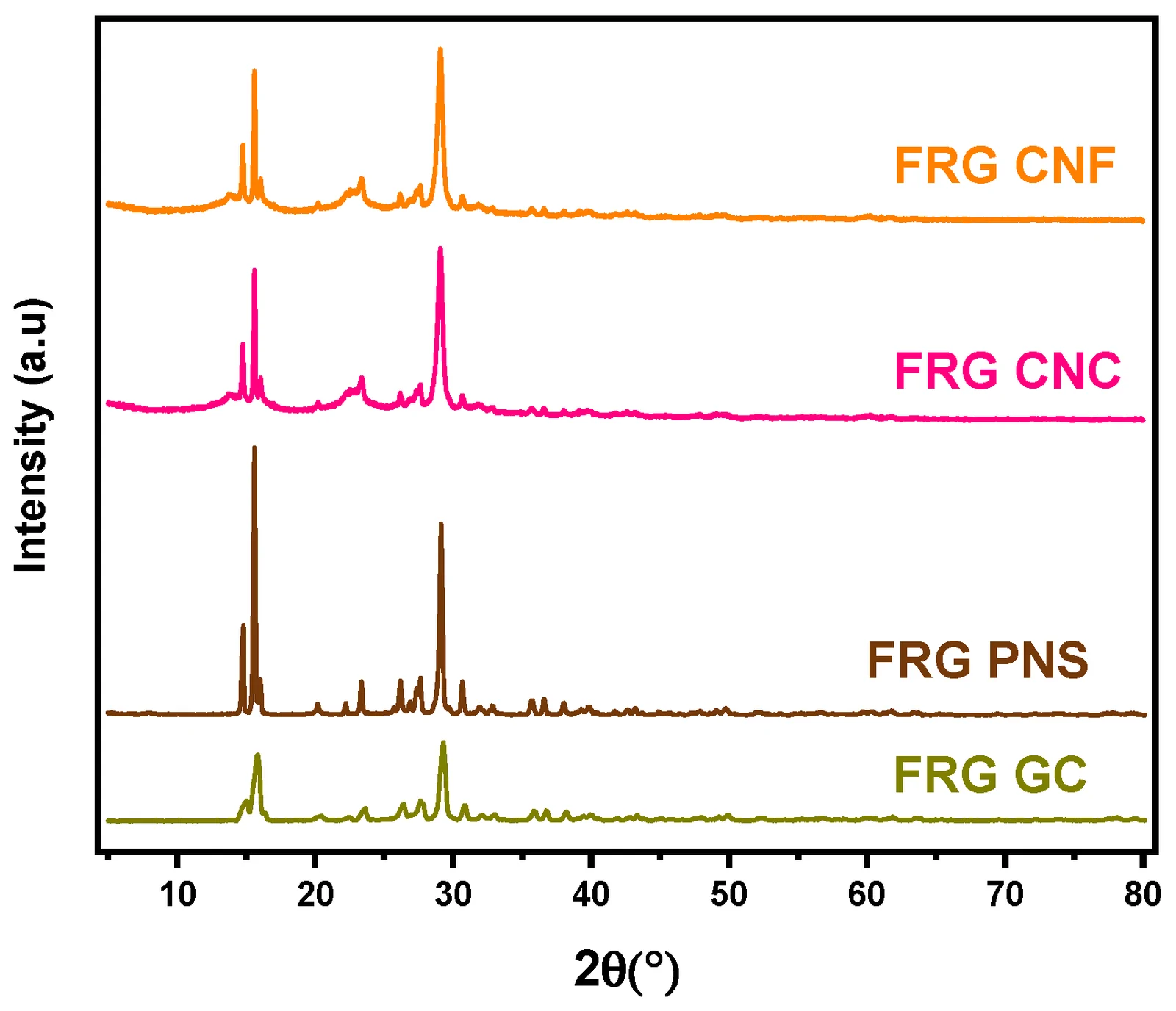
Comparative XRD patterns of the hybrid flame-retardant grenade (FRG) systems formulated with commercial (CNC and CNF) and waste-derived (PNS and GC) celluloses.

The crystalline structure of the hybrid Flame-Retardant Grenade (FRG) systems was evaluated by X-ray diffraction to determine whether the assembly process modified the structural organization of the incorporated constituents. The diffractograms presented in Figure 16 and Figure 17 preserve the principal diffraction features of the individual components, indicating that the preparation procedure did not induce detectable crystalline phase transformations during the fabrication of the hybrid architectures. Figure 16 presents the XRD pattern of FRG GC. The typical diffraction signals corresponding to cellulose (GC-BM) and SiO_2_ within the 15–25° *2θ* range are absent, owing to the amorphous nature of both synthesized silica and processed cellulose. The sharp, intense diffraction peaks of APP and ADA mask these broad amorphous signals. However, their presence is confirmed by FTIR and TGA, verifying that the SiO_2_ and cellulose support are retained in the FRG. As shown in Figure 16, this behavior is consistently observed across all FRG systems. Although the overall diffraction patterns are similar, subtle differences in peak width are observed depending on the cellulose source. The FRG-CNC and FRG-CNF formulations exhibit broader cellulose reflections than the corresponding FRG-GC and FRG-PNS systems. This behavior is consistent with the lower crystallinity and higher structural disorder previously identified for the waste-derived cellulosic materials, which retain residual amorphous constituents such as lignin following the extraction process. Conversely, the commercial nanocelluloses display sharper and more intense reflections, reflecting their higher crystalline order. No additional diffraction peaks associated with crystalline impurities or newly formed inorganic phases were detected after assembly of the hybrid systems. These results indicate that the fabrication procedure preserves the structural integrity of both the organic and inorganic constituents while producing homogeneous multi-component flame-retardant architectures. The structural characteristics identified by XRD complement the FTIR and TGA results, demonstrating that the FRG architectures maintain the intrinsic structural features of each constituent prior to evaluating their influence on the fire performance of the LDPE composites.

#### 3.3.4. TEM and SEM Analysis: Morphological Evidence of the FRG Assembly

**Figure 18:**
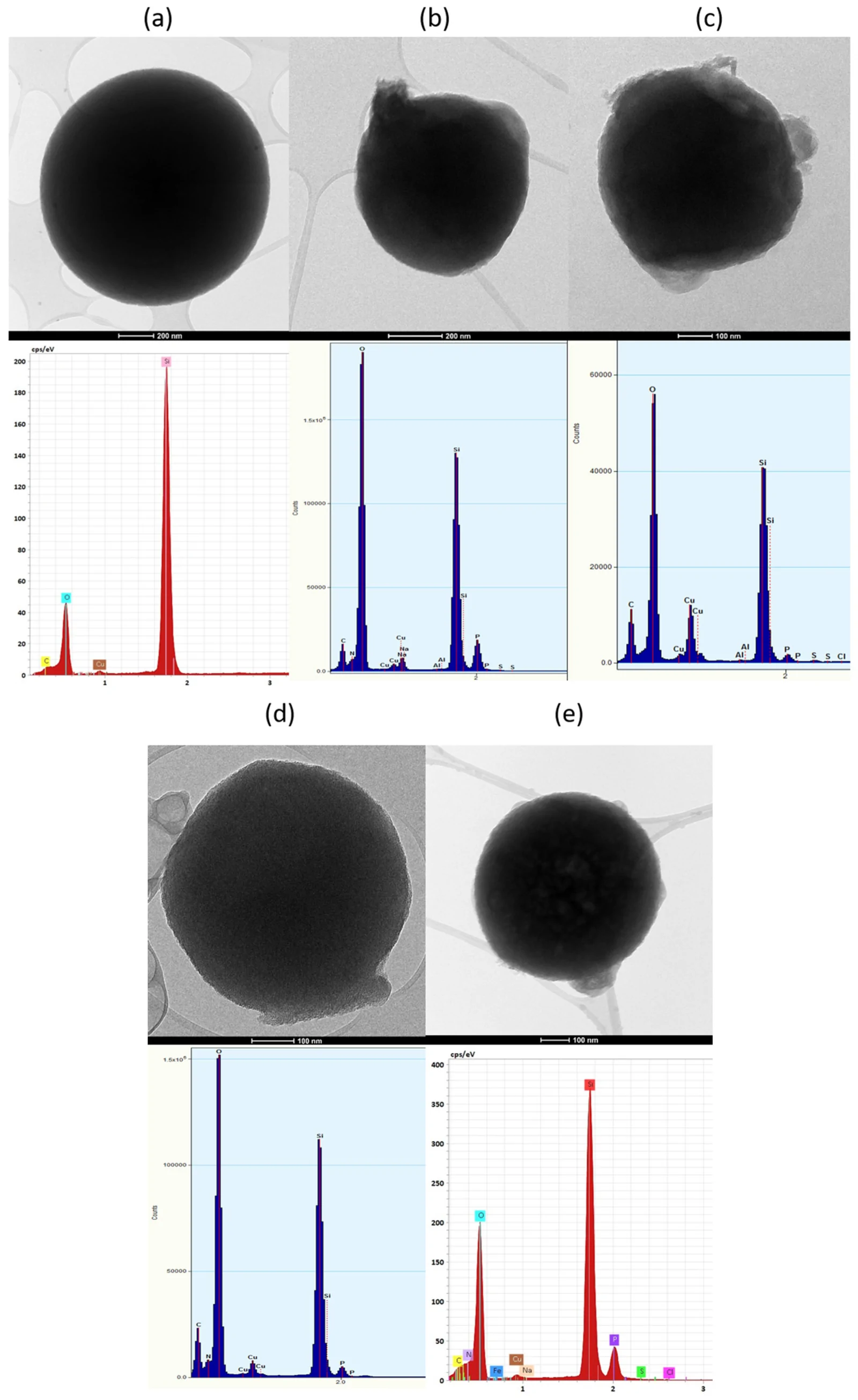
TEM micrographs of the synthesized amorphous SiO_2_ particles and the hybrid Flame-Retardant Grenade (FRG) systems: (**a**) synthesized SiO_2_; (**b**) FRG-CNC; (**c**) FRG-CNF; (**d**) FRG-PNS; and (**e**) FRG-GC.

Transmission electron microscopy (TEM) was employed to examine the morphology and spatial organization of the synthesized SiO_2_ particles within the hybrid Flame-Retardant Grenade (FRG) systems. Representative micrographs are presented in Figure 18, providing direct visualization of the microstructural organization achieved after assembly. The TEM images confirm that the synthesized SiO_2_ particles preserve their nearly spherical morphology after incorporation into the FRG architectures. These observations are consistent with the controlled synthesis of the amorphous silica previously characterized by XRD and with the narrow particle-size distribution determined by DLS, which yielded an average hydrodynamic diameter of approximately 533 nm. Figure 18b–e shows SiO_2_ core particles decorated with smaller particulates, likely formed by the recrystallization of APP and ADA on the surface and within the pores. This is supported by EDS analysis detecting Si, O, N, P, and C. The low carbon signal is attributed to ADA and small cellulosic debris co-deposited onto the SiO_2_ surface alongside APP and ADC.

**Figure 19:**
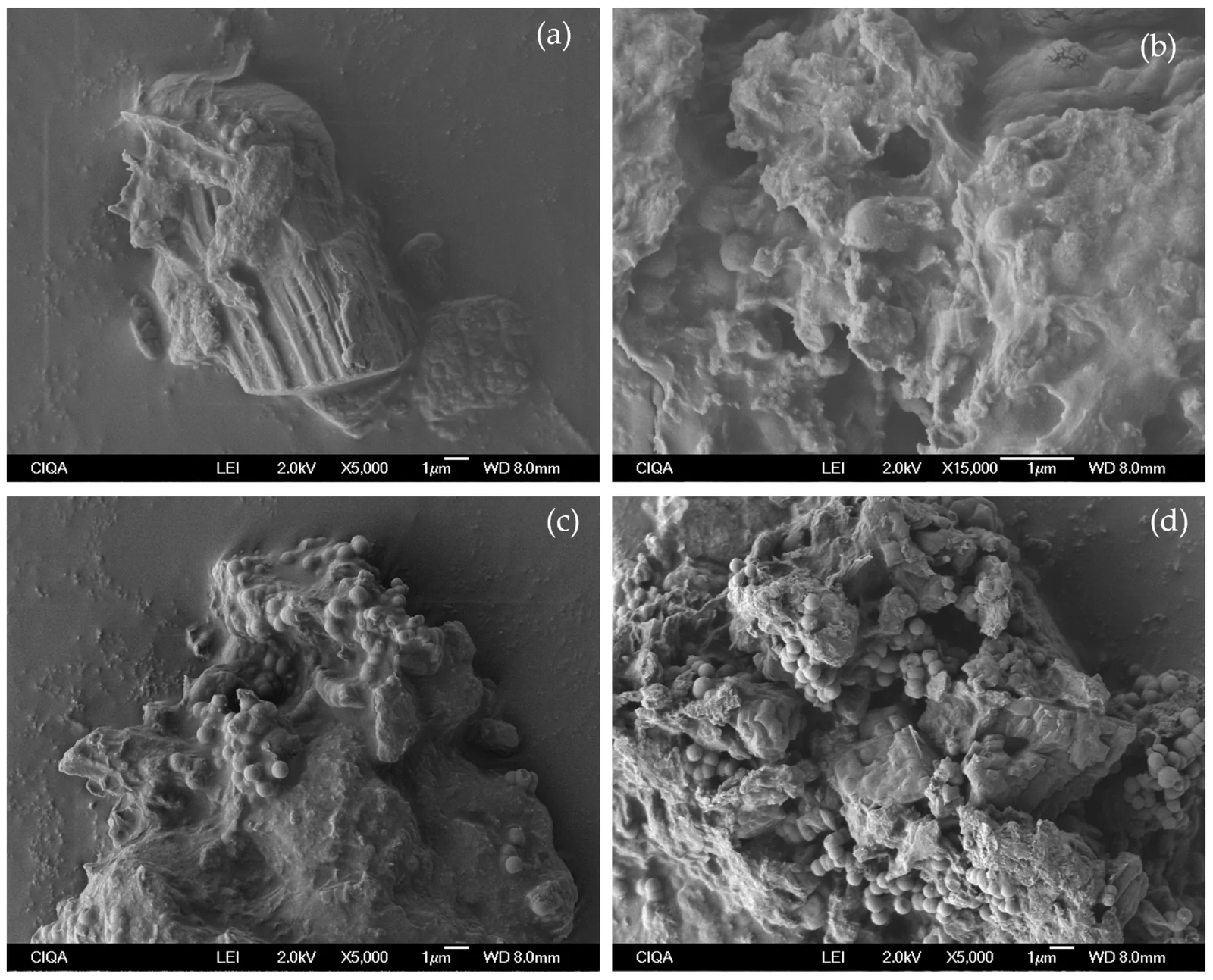
SEM micrographs of the FRG systems: (**a**) FRG-CNF, (**b**) FRG-CNC, (**c**) FRG-PNS and (**d**) FRG-GC.

Scanning electron microscopy (SEM) analysis corroborates the hierarchical assembly of the FRG architecture (Figure 19a–d). Differences in morphology particles are observed depending on the nature of the incorporated cellulose. In the commercial nanocellulose-based formulations (FRG-CNC and FRG-CNF), the characteristic rod-like morphology of cellulose nanocrystals and the entangled fibrillar network of cellulose nanofibrils remain distinguishable after assembly. The spherical SiO_2_ particles are observed in close proximity to these cellulosic structures, indicating a homogeneous spatial distribution of the inorganic phase throughout the hybrid architecture. In contrast, the waste-derived formulations (FRG-PNS and FRG-GC) exhibit a more heterogeneous morphology associated with the larger dimensions and irregular geometry of the extracted cellulosic fibers; nevertheless, densely embedded within the crevices and intimately anchored to the surface of this macro-structure are numerous uniform, sub-micron spherical assemblies. These spheres correspond to the surface-decorated “grenade” architectures (SiO_2_-ADA-APP) previously confirmed via TEM. This visual evidence unequivocally demonstrates a hierarchical support mechanism: the extensive cellulosic network acts as the primary macroscopic foundational support, effectively immobilizing the SiO_2_-ADA-APP system. By anchoring these active flame-retardant spheres across its highly irregular surface, the lignocellulosic scaffold prevents their macroscopic agglomeration. This hierarchical morphology may favor contact among the active components and the development of a protective residue, but it does not establish their initial dispersion within the melt-compounded LDPE matrix.

In conclusion, while both SiO_2_ and cellulose function as structural scaffolds for the active flame-retardant agents (APP and ADA), the cellulosic matrix ultimately acts as the primary foundational support for the entire hybrid architecture. The ultrasound treatment and aqueous dissolution of APP and ADA facilitate the homogeneous mixing of FRG components with porous SiO_2_ and cellulose particles, enabling physical anchoring within pores and onto surfaces, alongside electrostatic interactions. The morphological observations obtained by TEM and SEM complement the FTIR, XRD, and TGA analyses by confirming the uniform spatial organization of the organic and inorganic components prior to evaluating their influence on the combustion behavior of the LDPE composites.

### 3.4. Characterization of LDPE/FRG Composites Performance

#### 3.4.1. Thermal Stability and Transition Behavior (TGA & DSC)

**Figure 20:**
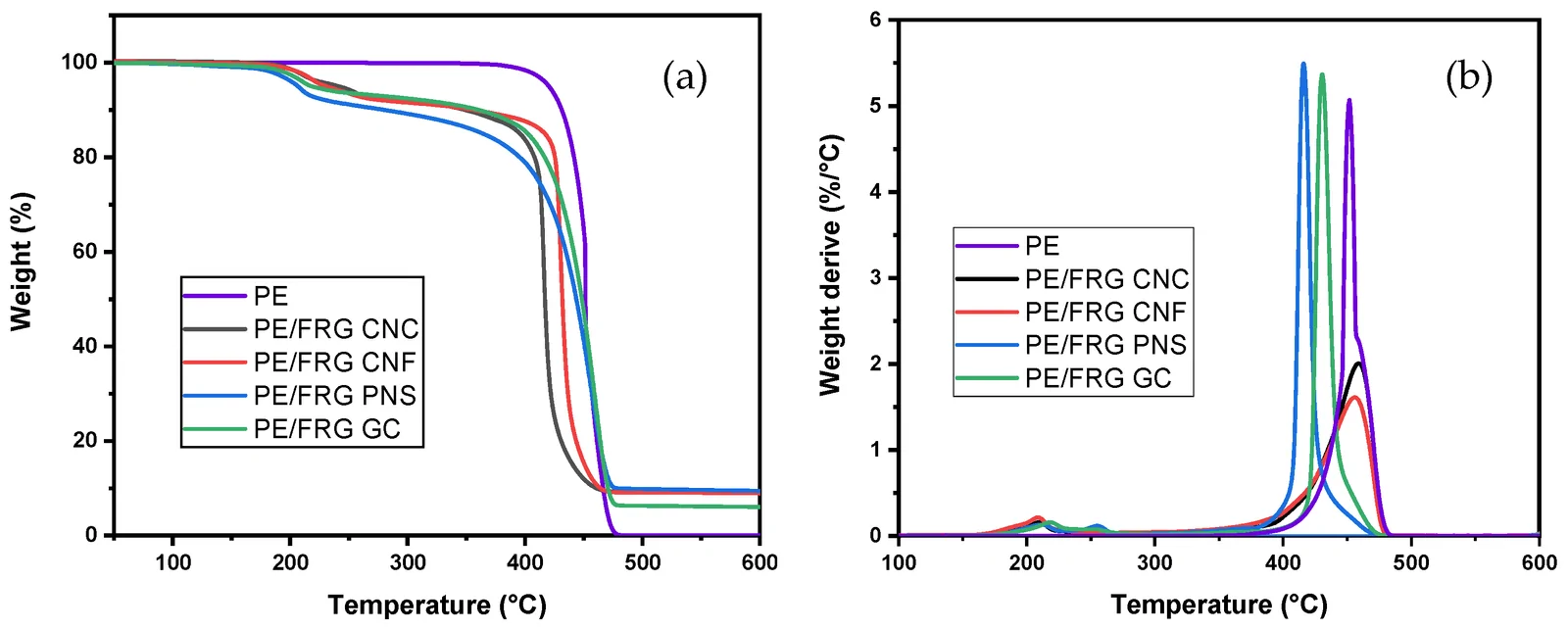
(**a**) TGA and (**b**) DTG thermograms of neat LDPE and the corresponding PE/FRG composites under a nitrogen atmosphere.

The thermal degradation behavior of neat LDPE and the corresponding LDPE/FRG composites was evaluated by thermogravimetric analysis (TGA) and derivative thermogravimetry (DTG). The thermograms presented in Figure 20 demonstrate that the incorporation of the FRG architectures substantially modified the degradation profile of the polyethylene matrix. Neat LDPE exhibited the highest onset thermal stability, with a temperature corresponding to 5 wt% mass loss (*T_5_%*) of 420 °C and a maximum degradation temperature (*Tmax*) of 451 °C. In contrast, all PE/FRG composites exhibited lower *T_5_%* values ranging from 206 to 241 °C. This earlier mass loss is associated with the intentional thermal activation of the intumescent flame-retardant components, primarily APP and ADA, which decompose before the degradation of the polyethylene matrix. This behavior is consistent with the proposed formation of acidic phosphorus-containing species that may promote cellulose dehydration and residue formation before the main degradation of polyethylene; TGA alone does not identify these species. Although the incorporation of the FRG systems reduced the initial thermal stability of the composites, differences were observed in the subsequent degradation behavior. The PE/FRG-CNC and PE/FRG-CNF composites exhibited *Tmax* values of 458 °C and 455 °C, respectively, slightly higher than those of neat LDPE, whereas PE/FRG-PNS and PE/FRG-GC exhibited *Tmax* values of 416 °C and 429 °C, respectively. The lower maximum degradation temperatures observed for the waste-derived formulations are consistent with the broader degradation profiles previously identified for the isolated cellulosic scaffolds, which retain residual lignin and other amorphous constituents. Despite the earlier onset of degradation, all PE/FRG composites generated substantially higher residual char than neat LDPE. While neat LDPE left only 0.2 wt% residue at 600 °C, the hybrid composites produced char yields ranging from 6.0 to 9.5 wt%, demonstrating enhanced solid residue retention during thermal degradation. This residue includes carbonaceous and inorganic constituents and is consistent with condensed-phase protection, but is not a direct measure of retained carbon or lignin carbonization.

**Figure 21:**
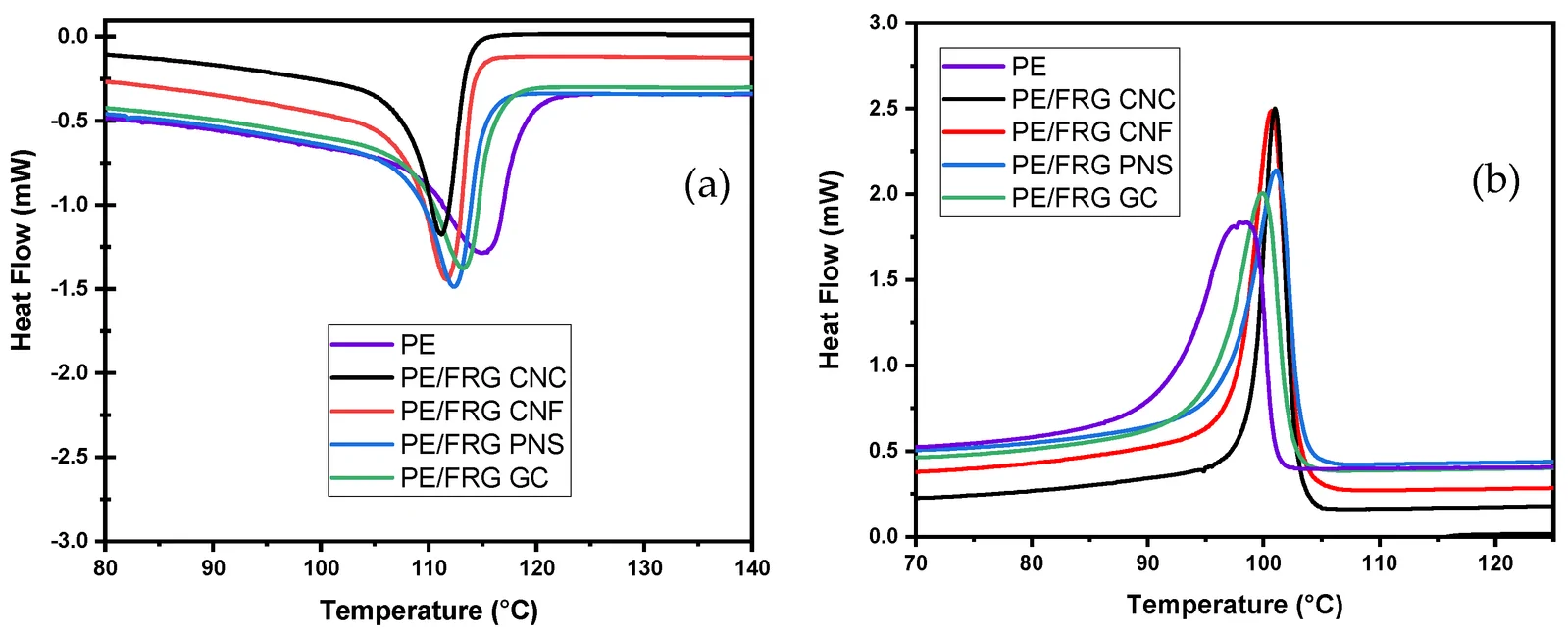
DSC thermograms of neat LDPE and PE/FRG composites: (**a**) second heating cycle and (**b**) cooling cycle.

Differential scanning calorimetry (DSC) was employed to evaluate the influence of the FRG architectures on the crystallization and melting behavior of the polyethylene matrix. The second heating and cooling thermograms are presented in Figure 21. During cooling, neat LDPE exhibited a crystallization temperature (*Tc*) of 98.1 °C. The incorporation of the FRG architectures shifted the crystallization exotherms toward slightly higher temperatures for all formulations, with *Tc* values of 99 °C for PE/FRG-GC, 100 °C for PE/FRG-CNF, and 101 °C for both PE/FRG-CNC and PE/FRG-PNS. This systematic increase suggests that the incorporated FRG particles act as heterogeneous nucleation sites, promoting the earlier onset of polyethylene crystallization during cooling. The melting behavior was only slightly affected by the incorporation of the hybrid architectures. The melting peak temperature (*T_m_*) decreased from approximately 115 °C for neat LDPE to values between 111 and 113 °C for the composites. Such small reductions in melting temperature have been widely reported for particulate-filled polyethylene systems and are generally associated with slight modifications in crystal morphology rather than changes in the crystalline phase itself. The degree of crystallinity (*Xc*) was calculated by normalizing the experimental melting enthalpy (*ΔHm*) to the actual polyethylene mass fraction in each formulation (*WPE* = 0.80), using 293 J g^−1^ as the melting enthalpy of 100% crystalline polyethylene. Neat LDPE exhibited a crystallinity of 30.58%. The incorporation of the FRG architectures generally maintained or slightly increased the crystallinity of the polymer matrix. PE/FRG-CNF (32.25%), PE/FRG-GC (32.01%), and PE/FRG-PNS (31.01%) exhibited crystallinity values comparable to or slightly higher than neat LDPE, indicating that the earlier crystallization observed during cooling was accompanied by similar or slightly improved crystalline development. In contrast, PE/FRG-CNC exhibited a lower crystallinity (26.89%) despite presenting one of the highest crystallization temperatures. This behavior suggests that, although the CNC-based architecture effectively promotes heterogeneous nucleation, the subsequent crystal growth may be partially restricted by the high surface area and rigid nature of the nanocrystalline cellulose, resulting in a modest reduction in the final crystalline fraction. Overall, the DSC results indicate that the incorporation of the FRG architectures modifies the crystallization behavior of polyethylene without substantially altering its melting characteristics. The slight increase in crystallization temperature together with the generally preserved crystallinity demonstrates that the hybrid flame-retardant architectures do not significantly disrupt the crystalline structure of the polymer matrix. Table 2 summarizes the DSC and TGA results discussed in the previous section.

#### 3.4.2. Flammability Performance of LDPE/FRG Composites

The horizontal burning behavior of neat LDPE and the corresponding PE/FRG composites was evaluated using the UL-94 horizontal burning test. The test provides quantitative information regarding flame propagation, dripping behavior, and horizontal burning rate. The experimental results are summarized in Table 3. Neat LDPE exhibited the characteristic behavior of an unmodified polyolefin under direct flame exposure. The specimen burned continuously until complete consumption, accompanied by intense flaming melt dripping that ignited the cotton indicator positioned beneath the sample. As a result, neat LDPE underwent complete burnout without measurable residual char, although due to its burning rate, it is classified as HB according to the UL 94 standard. The incorporation of 20 wt% of the FRG architectures substantially modified the combustion behavior of the polyethylene matrix. All PE/FRG composites completely suppressed flaming melt dripping during the horizontal burning test. In addition, all formulated systems were classified as HB according to the standard. Table 3 summarizes the UL 94 test results for all materials.

The burning behavior was influenced by the type of cellulosic scaffold incorporated into the FRG architecture. Among all formulations, PE/FRG-PNS exhibited the lowest linear burning rate (18.3 mm min^−1^), followed by PE/FRG-GC (19.4 mm min^−1^). These values were considerably lower than those measured for the commercial nanocellulose-based systems, PE/FRG-CNF (26.82 mm min^−1^) and PE/FRG-CNC (27.29 mm min^−1^). Similarly, clear differences were observed in the residual char yield obtained from the cone calorimeter test. PE/FRG-PNS produced the highest post-combustion residue (7.91 wt%), followed by PE/FRG-CNC (6.4 wt%), PE/FRG-GC (4.75 wt%), and PE/FRG-CNF (4.4 wt%). Although the amount of residual char does not directly determine the UL-94 classification, the higher residue retained after combustion is consistent with condensed-phase protection; the total residue includes both carbonaceous and inorganic FRG-derived components. Overall, the UL-94 HB results demonstrate that the incorporation of the FRG architectures significantly improves the horizontal burning performance of LDPE by eliminating flaming melt dripping, reducing the burning rate, and promoting residual char formation. Among the evaluated formulations, the waste-derived systems, particularly PE/FRG-PNS, exhibited the best overall performance under horizontal flame exposure. The different cone-calorimetry residue yields of PNS (7.91 wt%) and GC (4.75 wt%), despite qualitative evidence of lignin in both, show that lignin presence alone is insufficient to explain their behavior. Residual lignin may contribute, while particle size and morphology, cellulosic crystalline order, and FRG component distribution may also affect contact with APP and the development and continuity of the protective layer. Their individual contributions cannot be isolated or quantified from the available results.

**Figure 22:**
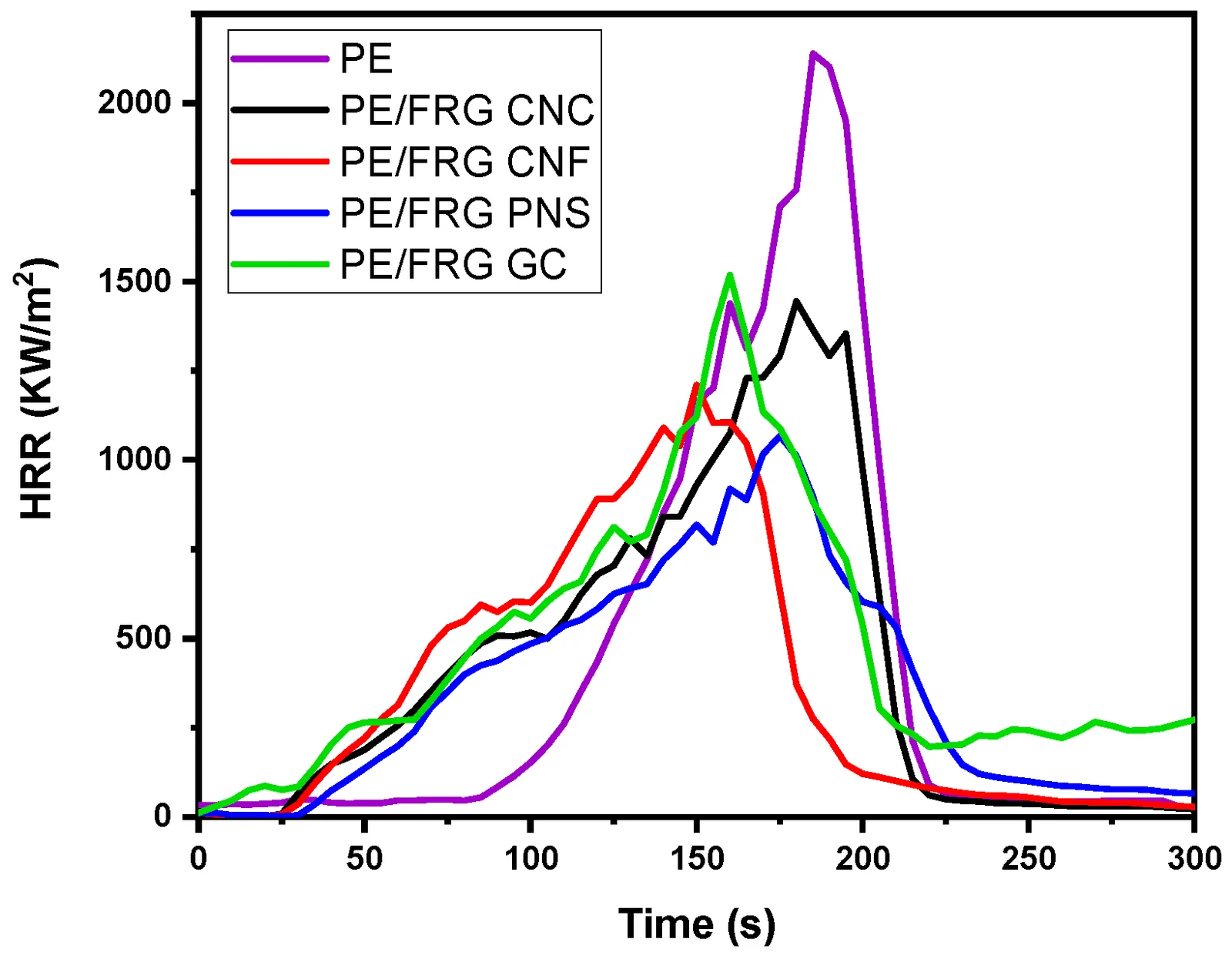
Heat Release Rate (*HRR*) curves as a function of time for neat PE and the corresponding PE/FRG hybrid composites by cone calorimeter test.

Figure 22 displays the heat release rate (*HRR*) curves from cone calorimetry testing of the PE/FRG composites. As shown in Table 4, neat PE exhibited a peak heat release rate (*PHRR*) of 2286 kW/m^2^; however, the addition of the studied FRGs provided flame retardant action across all formulations, reducing the *PHRR* to 1445, 1209, 1256, and 1518 kW/m^2^ for PE/GRF-CNC, PE/FRG-CNF, PE/FRG-PNS, and PE/FRG-GC, respectively. The most pronounced reductions were achieved with GRF-PNS and GRF-CNF, corresponding to a decrease of up to 48%. Although all composites initiated combustion earlier than neat PE, their lower total heat release *(THR*) values confirm effective flame-retardant performance (Table 4), as evidenced by the concurrent reduction in both *PHRR* and *THR* upon FRG incorporation.

**Figure 23:**
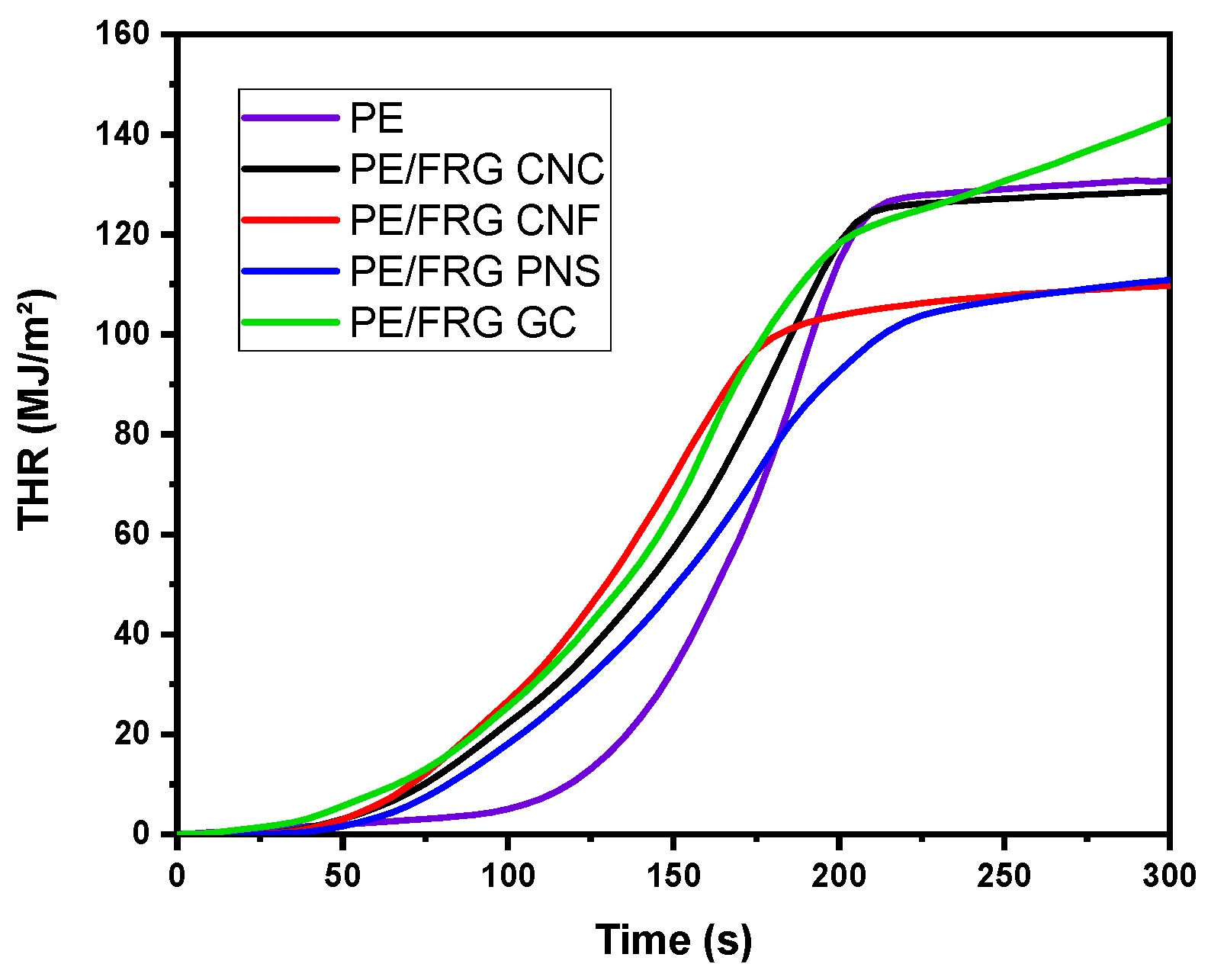
Total Heat Release (*THR*) profiles demonstrating the reduction in cumulative thermal output for the functionalized hybrid composite formulations.

Figure 23 illustrates the total heat release (*THR*) behavior of the PE/FRG composites in comparison to neat PE. The *THR* curves increase over time, following a trend similar to that of polyethylene; however, between 150 and 175 s, the PE/FRG-PNS and PE/FRG-CNF composites exhibit lower *THR* values than neat PE, consistent with their reduced *PHRR*. In contrast, the *THR* profiles of PE/FRG-GC and PE/FRG-CNC closely resemble those of neat PE. These cone calorimetry results demonstrate that the synthesized FRGs effectively retard PE combustion, with their flame-retardant efficiency depending on component composition and structural morphology.

**Figure 24:**
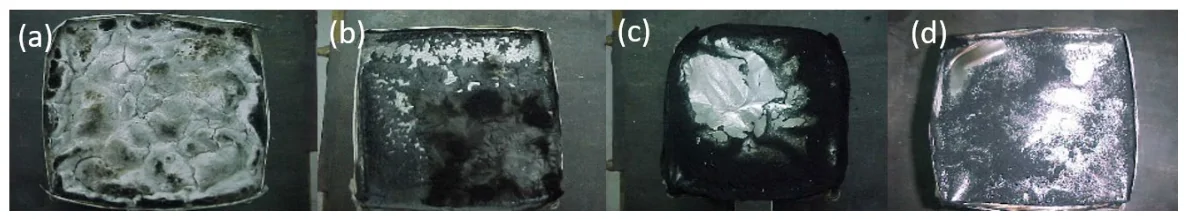
Macrostructural residues of PE/FRG composites monitored via cone calorimetry. PE/FRG PNS (**a**), PE/FRG CNF (**b**), PE/FRG CNC (**c**), PE/FRG GC (**d**).

Combustion residue morphology from cone calorimetry tests highlights distinct differences in fire behavior across the formulations. As depicted in Figure 24a, PE/FRG-PNS generates a continuous, expanded char layer that acts as an effective thermal and mass barrier. Figure 24b shows that PE/FRG-CNF also forms a continuous char, though it fails to span the entire sample surface owing to reduced residue formation relative to PE/FRG-PNS. Conversely, Figure 24c,d exhibit discontinuous, highly heterogeneous char structures with exposed regions, reflecting a diminished flame-retardant efficacy. The more continuous PNS residue compared with the heterogeneous GC residue suggests that residue organization, as well as its amount, contributes to the observed performance. These images do not allow the initial FRG dispersion within LDPE to be unequivocally reconstructed.

**Figure 25:**
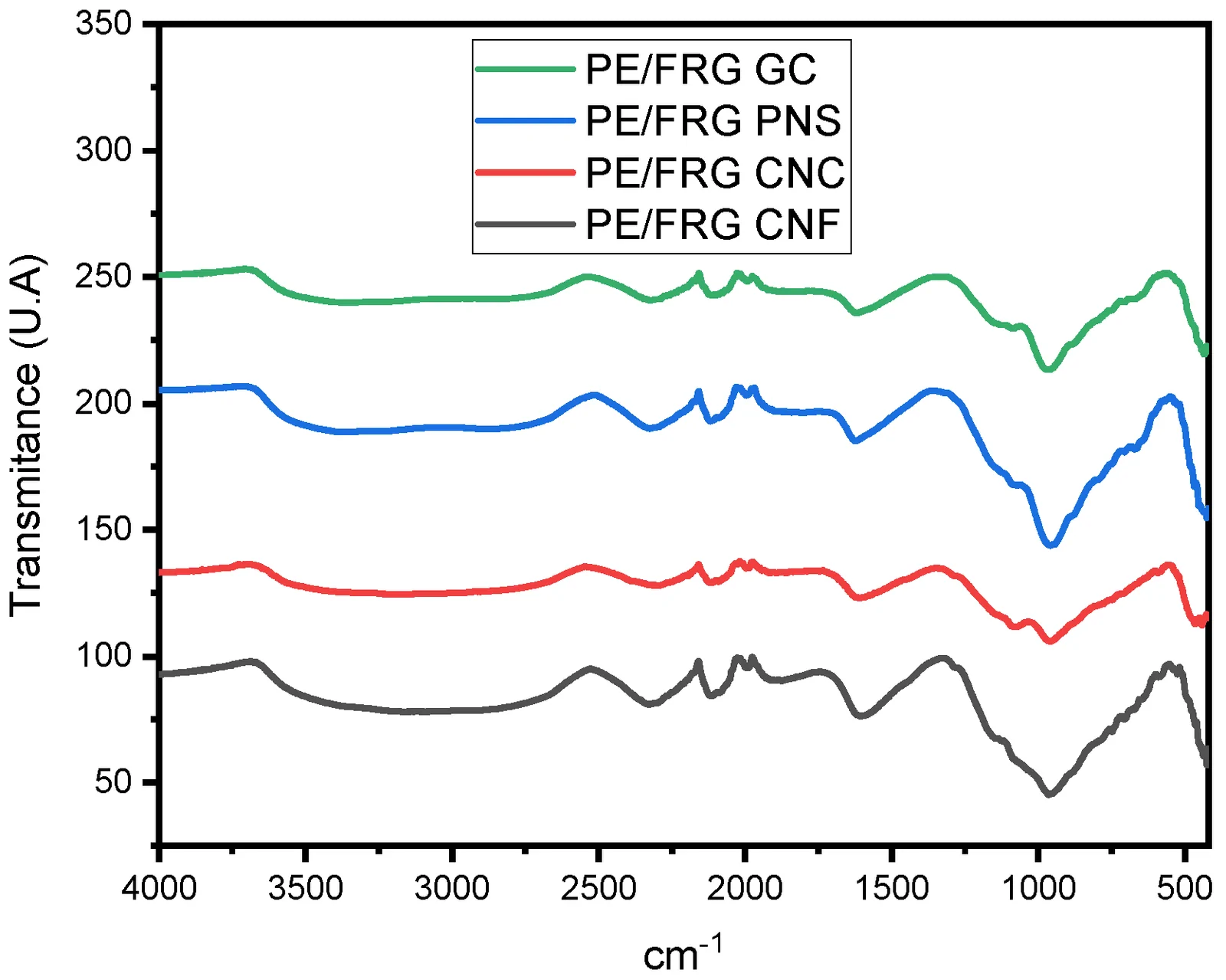
The FTIR spectra of the residues from the PE/FRG PNS, PE/FRG GC, PE/FRG CNC, and PE/FRG CNF.

The FTIR spectra of the residues from the PE/FRG PNS, PE/FRG GC, PE/FRG CNC, and PE/FRG CNF systems shown in Figure 25, similar absorption regions, although their recorded transmittance values differ. The broad band between 3600 and 3000 cm^−1^ is consistent with O–H stretching vibrations associated with hydroxyl groups and adsorbed water of SiO_2_ [35], whereas the band near 1620–1640 cm^−1^ may include the stretching vibrations of conjugated C=C bonds, C-N or aromatic rings derived from the pyrolysis of organic matter, APP and ADA. The fingerprint region exhibits a broad absorption envelope extending approximately from 1250 to 800 cm^−1^, with shoulders around 1150–1050 cm^−1^ and a dominant transmittance minimum near 950–970 cm^−1^. This envelope can tentatively be attributed to overlapping contributions from APP-derived phosphate species and the silica phase, consistent with the P–O/P–O–P vibrations reported for APP and its thermal residues, together with Si–O–Si contributions around 1100 cm^−1^ and Si–OH contributions near 950 cm^−1^ [35,36,37]. Accordingly, the minimum near 960 cm^−1^ cannot be assigned exclusively to P–O–P vibrations and does not establish the formation of P–O–Si or Si–O–P linkages. The available spectra also do not permit an unambiguous assignment of P–O–C structures. The feature near 2350 cm^−1^ may arise from atmospheric CO_2_ and background contributions associated with the measurement conditions [38]. Overall, the spectral profiles are consistent with residues containing phosphate species, silica, and hydroxyl-bearing species.

**Figure 26:**
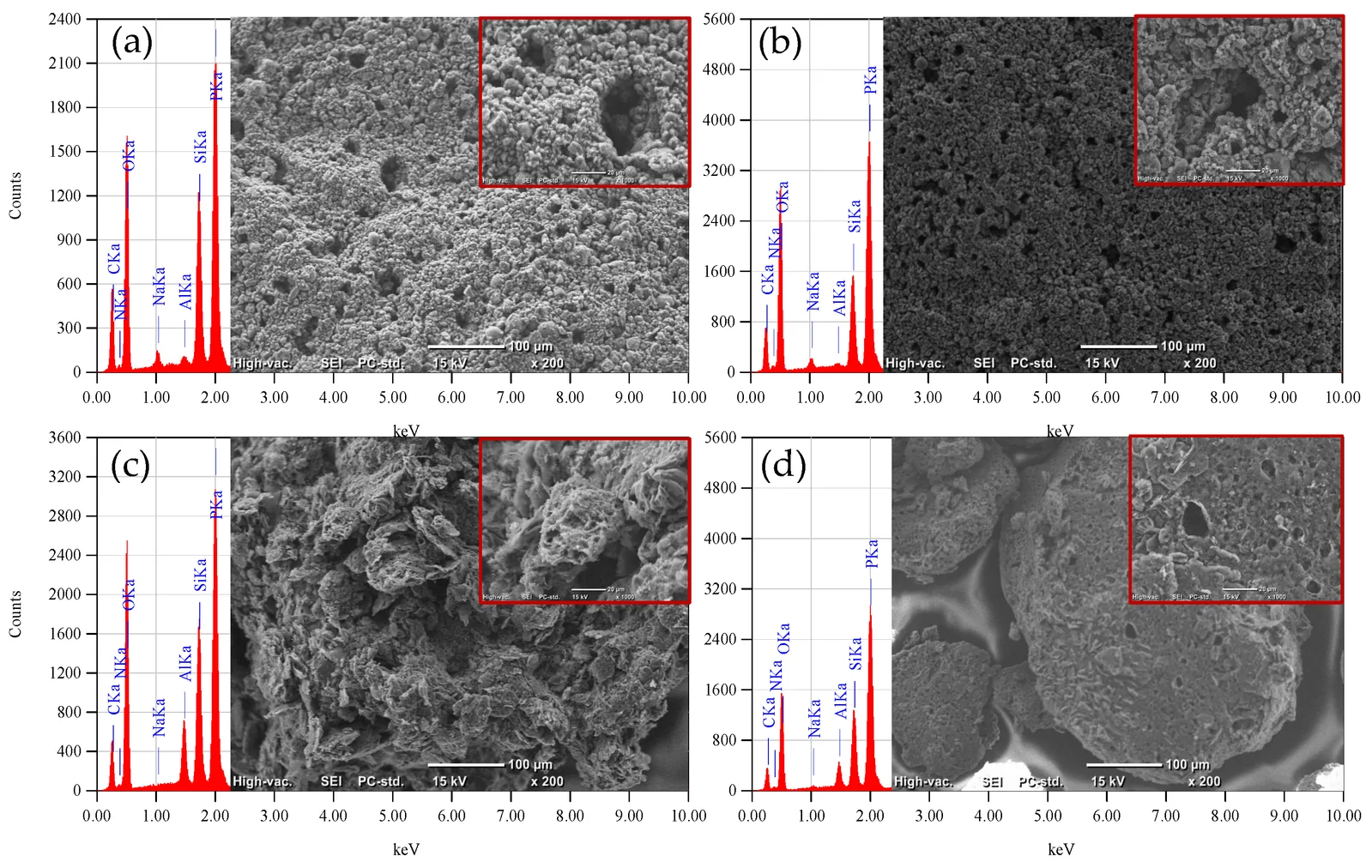
The SEM and EDS of the residues from (**a**) PE/FRG PNS, (**b**) PE/FRG GC, (**c**) PE/FRG CNF, and (**d**) PE/FRG CNC.

All four systems exhibit heterogeneous residues with cavities and different surface textures. PE/FRG PNS (Figure 26a) displays a relatively continuous granular surface within the examined field, with localized cavities. PE/FRG GC (Figure 26b) exhibits a finer granular texture and discontinuous surface. In contrast, PE/FRG CNF (Figure 26c) shows irregular aggregates with lamellar-like regions and interparticle spaces, whereas PE/FRG CNC (Figure 26d) exhibits larger fragments with porous surfaces and gaps between them. These differences indicate that the nature of the cellulosic scaffold influences the final residue morphology.

The corresponding EDS spectra show C and O signals, together with clearly identifiable P and Si signals, consistent with the retention of APP- and silica-derived components in the analyzed regions after combustion. Their coexistence supports the formation of a residue containing carbonaceous and inorganic components and is consistent with a protective contribution in the condensed phase [7]. The presence of cavities is consistent with the generation and release of volatiles during the thermal transformation of the material and with the formation of an intumescent structure. However, this morphology does not identify the gas composition or establish that the gases originate exclusively from ADA. A distinction must be made between the participation of gases in the expansion of a protective layer and a demonstrated flame-retardant action in the gas phase of the flame. The former may promote a barrier whose protective effect occurs in the condensed phase [39]. The potential role of ADA as a blowing agent is supported by literature reports of N_2_ and CO among its thermal decomposition products, together with isocyanic acid or ammonia depending on the temperature [40]. These literature findings support the ability of ADA to generate gases; however, gaseous products from the complete LDPE/FRG formulations were neither directly identified nor quantified. Accordingly, the observed porosity can be regarded as morphological evidence consistent with ADA participation in residue development. The FTIR and SEM/EDS analyses of the post-combustion residues confirm the simultaneous retention of both phosphorus- and silicon-based species within the condensed residue. However, as noted from the FTIR spectral envelopes (Figure 25), the overlapping signals in the 1250–800 cm^−1^ region cannot unequivocally confirm the formation of specific covalent P–O–Si or P–O–C linkages, nor do post-combustion SEM/EDS maps prove a completely uniform atomic-scale dispersion across the entire residue matrix. Instead, the elemental co-existence of P and Si indicates that SiO_2_ functions predominantly as a physical scaffold and rheological regulator during pyrolysis. During combustion, as the polyolefin matrix degrades and APP/cellulose components undergo intumescent crosslinking, the mesoporous SiO_2_ particles remain suspended within the transient viscous melt. These thermal-resistant inorganic spheres increase the local melt viscosity and act as physical frameworks that reinforce the thin cell walls of the expanding carbonaceous char. This physical scaffolding action prevents wall thinning, minimizes cell coalescing, and delays macroscopic char collapse or tearing under thermal convection. Consequently, the presence of SiO_2_ significantly contributes to the structural integrity and densification of the protective layer, explaining the complete elimination of flaming melt-dripping in UL-94 testing and the flattening of the heat release rate (*PHRR*) profiles. The lower horizontal burning rate of the pecan nutshell-derived formulation and the reduction in the peak heat release rate of the composites are consistent with this interpretation, although they do not allow the contributions of individual components to be quantitatively distinguished.

#### 3.4.3. Comprehensive Interpretation of the Fire-Retardant Mechanisms

The combined results obtained from thermogravimetric analysis (TGA), horizontal burning (UL-94 HB), cone calorimetry, and the structural characterization techniques provide a comprehensive understanding of the flame-retardant mechanism developed by the hybrid Flame-Retardant Grenade (FRG) architectures. Rather than acting through a single process, the experimental evidence suggests that the improved fire performance results from the interaction of the individual FRG constituents, which promote condensed-phase carbonization while simultaneously reducing heat release during combustion. The first stage of the protective mechanism is initiated by the early thermal decomposition of APP and ADA, as previously identified in the TGA analyses. The lower onset degradation temperatures observed for the PE/FRG composites compared with neat LDPE are consistent with the intentional activation of the intumescent system before the degradation of the polymer matrix. APP-derived acidic species are proposed to promote cellulose dehydration and charring, consistent with the thermal results and residue characterization rather than direct identification of the reaction pathway. ADA may act as a blowing agent during development of the protective residue. The nature of the cellulosic scaffold also plays an important role in the efficiency of the condensed-phase protection. The waste-derived formulation of PE/FRG-PNS exhibited higher residual char yields after thermal degradation and lower horizontal burning rates than the commercial nanocellulose-based systems. These observations are consistent with the structural characterization presented in previous sections, which demonstrated that the extracted agricultural fibers retain residual lignin after the extraction process. Residual lignin may contribute to carbonaceous residue formation, but its content was not quantified. The PNS–GC differences in residue retention and continuity therefore require a multifactorial interpretation involving composition, particle morphology and size, crystalline order, and component distribution; the contribution of each factor remains unresolved. Furthermore, the synthesized amorphous SiO_2_ particles are also expected to contribute to the structural stability of the protective layer. The observed organization within the FRG powders may facilitate contact between silica, APP, and the cellulosic scaffold. Recent investigations on high-temperature protective layers formed during polymer pyrolysis have emphasized that inorganic oxides (SiO_2_ and silicates) play a crucial dual role in char stabilization: controlling the high-temperature structural evolution of the residue and enhancing its physical barrier effect [41]. In the present hybrid FRG system, the functional interaction between the inorganic (SiO_2_, APP derivatives) and carbonaceous (cellulose/lignin char) phases can be understood through a physical reinforcement mechanism: mesoporous SiO_2_ acts as an inert high-temperature framework. During combustion, the thermal decomposition of ADA generates gaseous products that promote bubble nucleation and expansion, while APP promotes dehydration and carbonization of the cellulosic phase. The resulting expanded carbonaceous structure can retain the inorganic SiO_2_ phase within the developing char, as suggested by the SEM/EDS analysis. The presence of SiO_2_ may reinforce the cellular char structure, limiting bubble coalescence and reducing structural collapse and cracking [42]. Moreover, the accumulation of the inorganic phase within or near the exposed char surface can contribute to the formation of a thermally insulating protective layer, thereby reducing heat and mass transfer between the flame and the underlying polymer. This inorganic–carbonaceous layer may also increase the resistance to oxygen transport and retard the oxidative degradation of the underlying char at elevated temperatures [7,8]. The effectiveness of the FRG architecture is further reflected in the cone calorimetry results. Compared with neat LDPE, all hybrid composites exhibited a pronounced reduction in the peak heat release rate (*pHRR*) together with a broader heat release profile. Such behavior is characteristic of condensed-phase flame-retardant systems in which the protective residue progressively limits heat transfer from the external flame to the underlying polymer. The formation of this barrier reduces the degradation rate of the matrix, thereby decreasing the intensity of combustion. Similarly, the Total Heat Release (*THR*) curves reveal that the FRG formulations reduce the cumulative thermal energy released during combustion. This behavior is consistent with a protective residual barrier.

The lower burning rates observed during the UL-94 HB test together with the increased char yields and reduced heat release collectively support the effectiveness of the hybrid FRG architecture in improving the fire performance of LDPE. Overall, the experimental results suggest that the flame-retardant behavior of the FRG systems arises from the complementary functions of their individual constituents. Among the investigated formulations, the waste-derived systems, particularly PE/FRG-PNS, exhibited the most favorable balance between carbonization efficiency, burning resistance, and heat release reduction, demonstrating the potential of agricultural residues as sustainable components for advanced flame-retardant polyethylene composites.

#### 3.4.4. Mechanical Properties (Tension & Flexion)

The influence of the hybrid Flame-Retardant Grenade (FRG) architectures on the mechanical performance of LDPE was evaluated under uniaxial tensile loading and three-point bending. The tensile strength, elongation at break, flexural strength, and flexural modulus are summarized in Table 5.

The tensile results demonstrate that the incorporation of 20 wt% of the FRG architectures modified the mechanical behavior of the polyethylene matrix. Neat LDPE exhibited the typical response of a ductile thermoplastic, with a tensile strength of 14.90 MPa and an elongation at break of 566.30%. After incorporation of the hybrid flame-retardant systems, all composites exhibited a marked reduction in ductility, with elongation at break decreasing to values between 28.38% and 44.70%. This behavior is commonly observed in particle-filled polyethylene composites and is attributed to the restriction of polymer chain mobility imposed by the dispersed solid phases. The tensile strength was also influenced by the nature of the incorporated cellulosic scaffold. Among the evaluated formulations, PE/FRG CNC exhibited the lowest tensile strength (4.68 MPa), whereas PE/FRG CNF, PE/FRG PNS, and PE/FRG GC retained significantly higher values of 9.81 MPa, 9.80 MPa, and 9.10 MPa, respectively. These results indicate that the waste-derived formulations preserved a larger fraction of the mechanical resistance of the polymer matrix than the CNC-based system. Although the present study does not directly evaluate the fracture surfaces or filler dispersion, the improved tensile performance of the PE/FRG PNS and PE/FRG GC composites compared with PE/FRG CNC suggests that the morphology and chemical composition of the waste-derived cellulosic scaffolds may favor a more effective stress transfer within the composite. Additional interfacial characterization would be required to establish the specific mechanisms responsible for this behavior. The flexural properties exhibited a different trend. PE/FRG CNF showed the highest flexural performance among all formulations, with a flexural strength of 10.52 MPa and a flexural modulus of 332.00 MPa, both exceeding those of neat LDPE (9.70 MPa and 278.67 MPa, respectively). The enhanced flexural response is consistent with the fibrillar morphology of CNF, which has been widely reported to improve stiffness in polymer composites through its high aspect ratio and reinforcing capability. In contrast, PE/FRG CNC exhibited the lowest flexural strength (4.85 MPa) and flexural modulus (178.67 MPa). The waste-derived composites PE/FRG PNS and PE/FRG GC presented intermediate behavior, exhibiting identical flexural strengths of 6.40 MPa and flexural moduli of 182.50 MPa and 180.00 MPa, respectively. Although these values are lower than those of neat LDPE, they remain substantially higher than the tensile performance observed for the CNC-based formulation. The differences observed between the tensile and flexural responses indicate that the geometry of the reinforcing phase plays an important role in the mechanical behavior of the composites. The fibrillar morphology of CNF appears to provide greater reinforcement under bending, whereas the larger particles associated with the waste-derived cellulosic scaffolds primarily act as particulate fillers rather than continuous reinforcing networks. Overall, the incorporation of the FRG architectures produced the expected compromise between flame retardancy and mechanical performance. While all formulations exhibited reduced ductility compared with neat LDPE, the waste-derived composites PE/PNS and PE/GC maintained tensile properties comparable to those of PE/CNF while simultaneously providing superior flame-retardant performance, as demonstrated by the thermal and flammability analyses. These results indicate that agricultural waste-derived cellulosic scaffolds constitute promising sustainable alternatives for the development of multifunctional flame-retardant polyethylene composites.

## 4. Conclusions

In this study, sustainable hybrid flame-retardant architectures were successfully developed by integrating waste-derived cellulosic fractions from pecan shell and guayule as well as commercial nanocellulose systems into a low-density polyethylene (LDPE) matrix. The proposed Flame-Retardant Grenade (FRG) concept enabled the combination of ammonium polyphosphate (APP), azodicarbonamide (ADA), nano-SiO_2_, and cellulose-based carbon sources, providing a multifunctional approach to improve the fire response of polyethylene composites. The findings demonstrate that minimally processed agricultural wastes (pecan nutshell and guayule) effectively rival or outperform highly purified commercial nanocelluloses as carbon sources in intumescent systems. 

The assembly of ammonium polyphosphate (APP), azodicarbonamide (ADA), and mesoporous SiO_2_ onto the lignocellulosic scaffolds triggered an early intumescent activation during thermal degradation. This coordinated mechanism completely suppressed hazardous flaming melt-dripping in UL-94 HB testing and produced protective residues containing carbonaceous and inorganic constituents with char yields up to 9.5 wt% at 600 °C. Cone calorimetry analysis confirmed a drastic flattening of the peak heat release rate (*PHRR*) profiles, achieving up to a 45% reduction in *PHRR* of PE/FRG PNS relative to neat LDPE, supporting a protective contribution in the condensed phase. Residue FTIR and SEM/EDS support the retention of phosphate and silica containing constituents, without establishing specific phosphorus silicon bonds. ADA-mediated expansion remains a proposed contribution; gas-phase dilution or flame inhibition was not demonstrated in the complete formulations.

The pecan nutshell formulation (PE/FRG-PNS) exhibited the best overall balance of fire resistance and charring efficiency among the evaluated systems, associated with greater residue retention and a more continuous protective layer. Residual lignin is a possible contributing factor, rather than a demonstrated cause; compositional and structural contributions could not be individually quantified. While the incorporation of these rigid multifunctional particles predictably restricted matrix chain mobility, the hybrid composites maintained functional tensile strength. Ultimately, this FRG platform validates a viable circular-economy framework, successfully upcycling low-value biomass into high-performance, halogen-free flame retardants for polyolefins.

## Figures and Tables

**Table 1:** Melt-compounded formulations, their assigned nomenclature, and the multi-component mass distribution of the LDPE/FRG hybrid composites.

Sample ID	PE (wt%)	FRG (wt%)	Cellulose Type (Source)	APP (wt%)	ADA (wt%)	SiO_2_ (wt%)	Cellulose (wt%)
PE	100	0	None	0	0	0	0
PE/FRG CNC	80	20	Commercial Nanocrystals	6.25	6.25	1.25	6.25
PE/FRG CNF	80	20	Commercial Nanofibrils	6.25	6.25	1.25	6.25
PE/FRG PNS	80	20	Pecan Nutshell Cellulose	6.25	6.25	1.25	6.25
PE/FRG GC	80	20	Guayule Cellulose	6.25	6.25	1.25	6.25

**Table 2:** Quantitative TGA and DSC thermal degradation and non-isothermal phase transition parameters for neat PE and hybrid FRG composites.

SAMPLE	TGA/DTG			DSC			
	T_5%_ (°C)	T_max_ (°C)	Char Yield at 600 °C (wt%)	T_C_ (°C)	T_m_ (°C)	∆H_m_ (J/g)	X_C_ (%)
PE	420	451	0.2	98.1	115	89.6	30.58
PE/FRG CNC	241	458	9.4	101	111	63	26.89
PE/FRG CNF	230	455	9.3	100	111	75.67	32.25
PE/FRG PNS	206	416	9.5	101	112	72.88	31.01
PE/FRG GC	218	429	6	99	113	75.02	32.01

**Table 3:** Flammability parameters of neat LDPE and PE/FRG composites obtained from the UL-94 horizontal burning test.

Sample ID	Linear Flame Velocity (mm/min)	UL-94 Classification Status
PE	27.1	HB
PE/FRG-CNC	27.29	HB
PE/FRG-CNF	26.82	HB
PE/FRG-PNS	18.3	HB
PE/FRG-GC	19.4	HB

**Table 4:** Flammability parameters of neat LDPE and PE/FRG composites obtained from the UL-94 horizontal burning test.

Sample	*TTI* (s)	*pHRR* (kw/m^2^)	*THR* (MJ/m^2^)	Char Residue (%)
PE	82	2286.01	129.47	0.144
PE/FRG CNC	25	1445.29	125.33	6.4
PE/FRG CNF	26	1209.79	103.71	4.4
PE/FRG PNS	30	1256.48	101.29	7.91
PE/FRG GC	27	1518.30	118.92	4.75

**Table 5:** Mechanical properties of neat PE and its hybrid composites.

	Tensil		Flexural	
	Tensile Strength (MPa)	Elongation at Break (%)	Flexural Strength (MPa)	Flexural Modulus (MPa)
PE	14.9	566.30	9.7	278.67
PE/FRG CNC	4.68	37.96	4.85	178.67
PE/FRG CNF	9.81	28.38	10.52	332.00
PE/FRG PNS	9.80	42.00	6.40	182.50
PE/FRG GC	9.10	44.70	6.40	180.00

## Data Availability

The data that support the findings of this study are presented in this article. Further inquiries can be directed at the corresponding author.
